# Unveiling distinct storage composition and starch properties in developing indica rice grains via transcriptional profiling and enzymatic activity analysis

**DOI:** 10.1016/j.csbj.2025.11.011

**Published:** 2025-11-07

**Authors:** Wichian Sangwongchai, Kwanjeera Wanichthanarak, Ammarin In-on, Supidcha Natee, Chamaiporn Champasri, Nutcha Sa-ingthong, Diane M. Beckles, Sakda Khoomrung, Maysaya Thitisaksakul

**Affiliations:** a Department of Biochemistry, Faculty of Science, Khon Kaen University, Khon Kaen 4002, Thailand; b Siriraj Metabolomics and Phenomics Center, Faculty of Medicine Siriraj Hospital, Mahidol University, Bangkok 10700, Thailand; c Siriraj Center of Research Excellence in Metabolomics and Systems Biology (SiCORE-MSB), Faculty of Medicine Siriraj Hospital, Mahidol University, Bangkok 10700, Thailand; d Department of Plant Sciences, University of California, One Shields Avenue, Davis, CA 95616, USA; e Department of Biochemistry, Faculty of Medicine Siriraj Hospital, Mahidol University, Bangkok 10700, Thailand; f Thailand Metabolomics Association, Bangkok, Thailand

**Keywords:** Rice, Oryza sativa L. ssp. indica, Developing grain, Gene expression profile, RNA sequencing, Starch, Glutelin

## Abstract

The starch and protein in rice grains determine their nutritional value, eating and cooking quality (ECQ), and potential applications as a biopolymer. Building on previously identified functional differences between two Thai indica cultivars, waxy RD6 and high-amylose RD57, we examined the transcriptional regulation of their storage polymers by profiling key starch-biosynthetic and storage-protein genes in developing endosperms at 7-, 14-, 21-, and 28-days post-anthesis (DPA). Major differentially expressed genes were cross-validated with enzymatic activities and grain starch and storage-protein composition. Transcriptome dynamics varied significantly between cultivars, and were predominant at 14 DPA, which coincided with the period of active expression of the starch biosynthetic genes, while for storage protein gene expression, it was strongest at 7 DPA. At 21 and 28 DPA, most starch and glutelin biosynthesis genes in RD6 showed stronger expression than those in RD57. However, the *GBSSI*-to-*SS* expression ratio was higher in RD57, which correlated with its higher amylose content and associated differences in starch properties compared to RD6. Gene co-expression network analysis identified potential regulators of these pathways, including transcription factors bZIP10-like and bZIP44-like, and the energy signaling SnRK2.10-like kinase. Binding motif predictions indicated a bZIP10-like and bZIP44-like association with several starch genes, warranting further investigation as potential targets for improving rice starch. Collectively, this study provides a comprehensive understanding of the regulation of storage biomolecule biosynthesis in the previously understudied indica rice, laying the groundwork for breeders to develop new varieties with improved ECQ and health-beneficial traits for users in the food and biomaterial industries.

## Graphical Abstract

**Figure:**
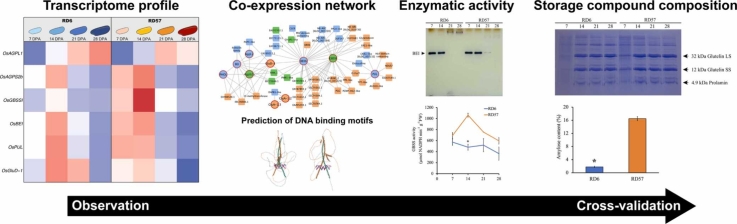

## 1 Introduction

Starch constitutes 80–90 % of the grain’s dry weight and is the world's most important source of dietary energy [1], [2]. Beyond serving as a primary staple, starch also determines rice eating and cooking quality (ECQ) [3], [4], [5] influencing consumers’ purchasing decisions and, in turn, its market price [5].

Rice ECQ, i.e., the hardness, elasticity, and stickiness of the cooked grain [3], [4], [5] is strongly linked to starch physicochemical and functional properties, such as swelling power, solubility, pasting, and thermal properties [4], [6], [7]. Starch functionality is largely determined by the relative proportion of the two starch glucans, the highly branched amylopectin and more linear amylose. Grain storage proteins are the second most common macromolecule found in rice grains but they negatively influence grain ECQ by limiting water availability for starch gelatinization upon cooking [8].

Our previous study of eleven Thai commercial rice cultivars [9], identified two with contrasting starch functional properties: RD57 a non-glutinous, high (i.e., 23.60 %) amylose rice cultivar, and RD6 a waxy, low (i.e., 1.65 %) amylose rice. These cultivars not only differ in amylose content but also in amylopectin fine structure and starch granule size, which in turn leads to differing starch functionality [9]. RD57 amylopectin, has a higher proportion of glucan chains of intermediate branch length, fewer short A- chains and, larger starch granules, which correlated with higher solubility, lower swelling power, higher pasting temperature and setback value, while requiring less energy for gelatinization compared to RD6 starch [9]. However, the genes and regulatory mechanisms underpinning differences in starch structure and hence functionality among these cultivars remain unexplored.

Starch biosynthesis is orchestrated by four main starch-synthesizing enzymes of which there are multiple isoforms [10], [11]. ADP glucose pyrophosphorylase (AGPase; EC 2.7.7.27), catalyzes the production of ADP-glucose, the primary substrate for starch biosynthesis. Starch synthases (EC 2.4.1.21) use ADP-glucose to elongate α-1,4 glucan chains in the starch molecule [11]. Starch synthase exists as two forms: granule-bound starch synthases (GBSS) and soluble starch synthase (SS) [10], [11]. Starch branching enzymes (SBE; EC 2.4.1.18), form new branch points by cleaving an internal α-1,4 linkage on a donor glucan and reattaching it to an acceptor chain via an α-1,6 glucan linkage [10], [12]. Starch debranching enzymes (DBE, EC 3.2.1.70), including isoamylase (ISA; EC 3.2.1.68) and pullulanase (PUL), hydrolyze α-1,6 glucan linkages to refine the structure of the polyglucan [10], [12], with ISA being necessary for the formation of the crystalline lamella in starch granules [10], [13]. Amylose biosynthesis is catalyzed by GBSS and SBE, resulting in a polyglucan with few branches, while amylopectin synthesis is mediated by various isoforms of SS (SSI, SSII and SSIII), SBE (SBEI, SBEIIa and SBEIIb), and DBE (ISA and PUL) [14], [15], [16].

Genes encoding these starch biosynthetic enzymes are primarily expressed between 7 and 28 days post-anthesis (DPA) in rice endosperm [11]. *GBSS* transcripts are highest at 7 DPA, whereas those for SS genes are expressed later in grain development, coincident with active amylopectin biosynthesis, and *GBSS* mRNA declines [11], [17]. In glutinous rice, amylose accumulation starts at 7 DPA, and remains constant until 42 DPA [11]. The coordinate regulation of GBSS and other starch biosynthesis enzymes determines the fine structure of amylose and amylopectin and thereby, the physicochemical properties of rice starch [11], [17], [18], [19].

Transcription factor (TF) families, such as the basic leucine zipper (bZIP) [20], MADS-box [21], MYC-like [22], NAC [23], and NF-Y [21], modulate the expression of starch metabolic genes. *OsbZIP58* regulates starch and amylose content and amylopectin structure by directly binding to the promoters of *OsAGPL3*, *Wx*, *OsSSIIa*, *OsBEI*, *OsBEIIb*, and *OsISA2*, and regulating their expression [20]. *OsMADS14*, works with NF-YB1, a nuclear factor-Y subunit, to activate the expression of *OsAGPL2* and *Wx* (*GBBSI*) in the endosperm [21]. *OsNAC25*, interacts with *OsNAC20* and *OsNAC26* to form regulatory loops that balance starch synthesis [23]. Altered *OsNAC25* expression causes chalky seed phenotypes and reduces starch content [23]. Notably, most of these regulatory studies have focused on the japonica rice ecotype [20], [21], [22], [23], while investigations in the indica rice ecotype are still limited [20], [21], [23].

Our goal was to address the knowledge gap related to the transcriptional regulation of storage product biosynthesis in indica rice and to identify genes that coordinately control their composition and accumulation. Because indica rice accounts for ∼75 % of global rice production this study has broad relevance. We therefore investigated differences in gene expression and the activities of enzymes in starch biosynthesis throughout grain filling period in two *Oryza sativa* L. ssp. indica cv, RD6 and RD57. These differences may underlie the distinct grain chemical compositions and starch properties observed between the two genotypes which are key to their ECQ [24]. We also examined the relationships among gene expression, enzymatic activity, and storage biomolecule composition to pinpoint potential transcriptional and post-translational regulatory mechanisms. A gene co-expression network analysis was also performed to identify putative regulators of starch and storage protein accumulation. This study aims to provide insights into the transcriptional networks regulating starch and storage protein biosynthesis in indica rice, offering potential targets for genetic improvement of grain quality.

## 2 Materials and methods

### 2.1 Plant materials and growth conditions

Rice grains of cultivars RD6 and RD57 were obtained from the Pathum Thani and Khon Kaen Rice Research Stations (Rice Department, Ministry of Agriculture and Cooperative, Thailand). Grains were germinated on petri dishes and transplanted into 64 8” plastic pots and placed in a net house in Completely Randomized Design under natural light conditions at the Faculty of Science, Khon Kaen University from September 2019 to January 2020 [9], [25]. Three replicates were grown for each developmental stage and for each cultivar, for transcriptome profiling, enzymatic activity assays, and storage protein composition analyses. A single panicle on the main stem of each rice plant reaching anthesis was tagged at the full heading stage. At 7,14, 21, and 28 DPA, superior grains on the apical primary branches of tagged panicle were cut and snap-frozen in liquid nitrogen. The frozen grains were stored at −70 °C until further analyses [26]. All panicles of each replicate were then harvested after the mature plants were left in the pots without water for 7 days. Thereafter, the air-dried mature seeds of the two rice varieties (Fig. S1) were used to determine grain starch composition [27], [28].

### 2.2 RNA extraction and Illumina sequencing

The total RNA was extracted from the RD57 and RD6 rice grains at 7, 14, 21, and 28 DPA with three biological replications each using the modified SDS-LiCl method [29]. The RNA ScreenTape assay of the Agilent 4150 TapeStation system (Agilent, USA) was used to check for the samples integrity. The extracted RNA with RIN values between 8.6 and 10.0 were then used for RNASeq library construction and subsequently sequenced by Illumina HiSeq 2000 SBS technology at Macrogen Genome Center (Seoul, Republic of Korea).

### 2.3 Quality assessment and processing of RNA sequencing data

Prior to data analysis, the quality of raw sequencing reads was assessed using FastQC (access 26 Mar 2024) [30], with the results subsequently summarized and visualized via MultiQC [31]. Low-quality reads were removed through two steps: (I) Fastp [32] was used to filter out reads and bases with a quality score (Q) less than 30; and (II) Trimmomatic [33] excluded adapter sequences, short reads (length < 100 bp), unpaired reads, and additional low-quality reads. rRNA sequences were identified and removed from the dataset using SortmeRNA [34]. Reference sequences for 5S rRNA and 5.8S rRNA were retrieved from the Rfam database [35], while the SILVA database [36] was used as an additional reference source for other rRNA sequences. Table S1 summarizes the number of reads before and after the filtering steps.

The processed reads were aligned to the *Oryza sativa* indica reference genome (https://www.ncbi.nlm.nih.gov/datasets/genome/GCA_000004655.2/) [37] using HISAT2 [38]. Approximately 90 % of the reads were concordantly mapped (Table S2). Pseudogenes, singletons, and discordant alignments were then filtered out to remove irrelevant data. Each sample was assembled separately using Cufflinks [39], guided by the reference genome. Cuffmerge was then employed to generate a transcript library from the 24 samples. Transcript sequences were retrieved from the genome reference using PASA [40], with guidance from the GTF library generated in the previous step. The completeness of the transcript structures was evaluated by BUSCO complete [41], which showed a completeness of 97.7 % (Table S3). Cuffquant estimated transcript abundance based on the transcript library, with each sample quantified independently. The resulting transcript abundances were normalized by Cuffnorm and scaled to Fragments Per Kilobase Million (FPKM). Expression values from transcripts sharing the same gene ID were merged to calculate gene-level expression. Genes with FPKM below 0.5 were excluded, and a gene must be detected in at least two replicates to be included. Missing values were imputed by averaging expression levels across replicates. Overall gene expression levels (Fig. S2), and gene distribution for each sample (Fig. S3) were examined prior to statistical analysis.

Genes involved in grain starch and storage protein biosynthesis were selected from published works [42], [43], [44]. A reciprocal BLASTn search [45] was performed to identify homologous genes from *O.sativa japonica* with criteria of 60 % identity, 70 % coverage, and an e-value threshold of 10^−10^. Additionally, genome annotation was conducted using eggNOG-mapper [46] against all eukaryotic genes in the eggNOG database. Genes related to the starch and storage protein biosynthetic pathways were selected for qPCR validation and further examination based on their functional roles.

### 2.4 Differentially expressed gene (DEG.) analysis and statistical analysis

Principal component analysis (PCA) was performed by Metabox 2.0 R package [47] to explore overall variations of gene expression profiles. DEG analysis was initiated by estimating the dispersion model and incorporating it into a generalized linear model (GLM) using all 24 expression datasets. The analysis compared RD57 to RD6 grains at the same developmental stage. Significantly expressed genes were identified based on these criteria: False Discovery Rate (FDR), controlled by the Benjamini-Hochberg procedure = < 0.05 and absolute log2 fold-change (Log2FC) > = 2. For this analysis, the edgeR-glmQLFit R package [48] was applied.

The temporal expression patterns of DEGs at one or more DPA were characterized using Short Time-series Expression Miner (STEM) [49]. Briefly, a set of unique temporal expression patterns was calculated. Each gene was assigned to the pattern that most closely matches its expression profile, as determined by the correlation coefficient. Significant temporal expression patterns for each genotype were then identified by evaluating the number of assigned genes through a permutation test (Bonferroni-corrected *p*-value < 0.001). Furthermore, significantly correlated temporal patterns between RD57 and RD6 were determined using a hypergeometric test (*p*-value < 0.05), based on the intersection of genes between the two patterns. Differences of gene expression profiles between patterns were also analyzed by correlation analysis. To visualize gene expression patterns in detail, heatmaps of expression levels and Log2FCs were generated, with clustering based on Euclidean distance.

Genes selected from each temporal expression pattern were subjected to co-expression analysis using Spearman’s method. We considered a *p*-value < 0.01 and an absolute correlation coefficient > 0.9 as the cutoff for significant correlations. A co-expression network was constructed using Cytoscape version 3.10.3 [50].

### 2.5 *In silico* analysis of TF binding motifs

The cis-element prediction analysis followed the method of In-on A. (2022) [51]. Briefly, upstream sequences (−1000 to −1) of starch and protein biosynthetic genes from *Oryza sativa* indica (ASM465V1) were retrieved from the RSAT database [52] using the sequence tool. A reciprocal BLASTp search (https://blast.ncbi.nlm.nih.gov/Blast.cgi) was performed against *Oryza* spp. included in CIS-BP database [53]. Both bZIP10 (Osl_28943) and bZIP44 were identified as members of the CIS-BP family ID F091_3.00. All position weight matrices (PWMs) associated with this TF family were retrieved from CIS-BP, and the upstream sequences were analyzed together using the RSAT pattern-matching tool. The second-order Markov model with default cutoffs was applied, and background frequencies were calculated from the *Oryza sativa* indica (ASM465V1) draft genome. TF–DNA binding interactions and 3D models were predicted using AlphaFold3 [54]. TF–DNA complex structures were filtered for DNA contact sites containing seven residues with the conserved motif SNRxSAxxSR [55]. Selected structures were refined using a High Ambiguity Driven protein-protein Docking approach (HADDOCK) [56], and hydrogen atoms were added with Reduce (https://github.com/cctbx/cctbx_project/tree/master/mmtbx/reduce). Ten HADDOCK models were generated for each TF–DNA pair, and the best docking models were selected by the lowest HADDOCK scores (< –400). The resulting binding motifs were extracted, and 3D structures were visualized using the PDB Mol 3D Viewer (https://www.rcsb.org/3d-view).

### 2.6 cDNA synthesis and quantitative real-time PCR

The 500 ng of total RNA was converted into complementary DNA (cDNA) by using ReverTra Ace qPCR RT Master Mix with gDNA remover (TOYOBO, Japan) according to the manufacturer’s recommendations. Quantitative real-time PCR analysis was performed using 0.5 µL of cDNA as template with THUNDERBIRD™ SYBR™ qPCR mix (TOYOBO, Japan) and performed by LightCycler®480 II system (Roche, Switzerland). The reaction was performed using 1X THUNDERBIRD™ SYBR™ qPCR mix and primer concentrations of 0.25 mM. The primer sequences were provided in Table S4. The relative expression level of each gene was calculated using ∆Ct method with three biological replicates. The Ct value of target genes was normalized by internal control and was reported as log_10_ relative expression level.

### 2.7 Native-PAGE and activity-gel staining of starch branching enzyme (SBE)

Enzyme extraction was carried out as previously described [57] with slight modifications. Three biological replicates of rice grains at 7, 14, 21, and 28 DAP were weighed and homogenized in an extraction buffer containing 100 mM Tricine-NaOH (pH 7.5), 8 mM MgCl₂, 2 mM EDTA, 12.5 % (v/v) glycerol, 1 % (w/v) polyvinylpyrrolidone (PVP), and 50 mM β-mercaptoethanol. The homogenates were centrifuged at 10,000 ×g for 20 min at 4 °C. The pellet and supernatant were used for SBE and GBSS activity analyses, respectively. Native-PAGE with 8 % (w/v) separating and 4 % (w/v) stacking gels were prepared as previously described [58]. Twenty-five micrograms of total protein from all samples were mixed with 1X native dye before loading. The electrophoresis was performed at 4 °C with 180 volts for 90 min using Mini-PROTEAN Tetra Cell (Bio-Rad, USA). The first gel was stained in 0.15 % (w/v) Coomassie Brilliant Blue for 1 h and subsequently destained with destaining solution to achieve the blue bands against clear background. The second gel was subjected to zymography or activity-gel staining. The detection process followed the previously described method [58], while reagents and method used for enzymatic activity assay were modified from Nishi et al. (2001) [59]. Gel was initially soaked in 2 % (w/v) glycogen (solubilized in 0.05 M HEPES-NaOH, pH 7.4) and was kept at 4 °C for 30 min. To detect starch branching enzymatic activity, 50 mM Glucose-1-phosphate, 2.5 mM AMP, 5 % (v/v) glycerol, and 55 units of rabbit muscle phosphorylase a were added. The catalytic activity of SBE was determined at 35 °C for 2 h prior to removal of reaction mixture. Gel was rinsed with distilled water and stained with iodine solution. The non-specific binding iodine was washed with distilled water until the dark blue bands were observed against clear background. The intense blue band indicates the high activity of SBE.

### 2.8 Measurement of granule-bound starch synthase (GBSS) activity in developing rice grains

GBSS activity was determined using the pellet derived from enzyme extraction in Section 2.7 [60]. The pellet were weighed, extracted in a buffer (100 mM HEPES-NaOH pH 7.5, 8 mM MgCl₂, 2 mM EDTA, 1 mM DTT, 12.5 % (v/v) glycerol, 5 % (w/v) PVPP), and centrifuged at 12000 ×g for 10 min at 4 °C. The starch granule pellet was suspended in 1 mL of extraction buffer twice (recorded as V1). The suspension of 0.1 mL (recorded as V2) was aliquoted into two tubes: a reference tube and an experimental tube, each containing 0.45 mL of reaction mixture (recorded as V3) (50 mM HEPES-NaOH pH 7.5, 15 mM DTT, 0.1 mL of enzyme extract, and 1.6 mM ADP-glucose). The experimental tube was then incubated at 30°C for 20 min. The tubes were then heated at 100°C for 1 min. After cooling to an ambient temperature, the reaction mixtures were centrifuged at 10,000 ×g for 10 min. A 0.3–0.4 mL supernatant (recorded as V4) was incubated with 0.2 mL of pyruvate kinase reaction mixture (50 mM HEPES-NaOH pH 7.5, 200 mM KCl, 10 mM MgCl₂, 4 mM phosphoenolpyruvate, and distilled water) and 2 µL of 1.2 U of pyruvate kinase, then heated at 100 °C for 1 min. The reaction mixture was centrifuged at 10,000 ×g for 10 min, then transferred to a new tube containing 0.4 mL of reaction mixture (50 mM HEPES-NaOH pH 7.5, 10 mM glucose, 20 mM MgCl₂, 2 mM NADP, and distilled water), and 2 µL of 1.4 U hexokinase. Then, 0.35 U Glucose-6-phosphate dehydrogenase was added to achieve the final mixture (recorded as V5). The reaction was incubated at 30°C for 10 min, and the absorbance was recorded at 340 nm. The GBSS activity was calculated using the following equation:

$$\mathrm{GBSS}\;\mathrm{activity}\;(µmol\;\mathrm{NADPH}\;\min^{-1}\;g^{-1}\mathrm{FW})\;=\;\frac{\mathrm{Abs}340}{6.22L}\;\times V5\times \;\frac{V1}{V2}\;\times \frac{V3}{V4}\times \frac{1}{m}\times \frac{1}{t}$$

### 2.9 Measurement of Amylose content

The Megazyme Amylose/Amylopectin Assay Kit (Megazyme, Ireland) was used to measure the amylose content in the 20 mg starch sample as instructed by the manufacturer. Three biological replicates were analyzed.

### 2.10 Measurement of grain storage protein with SDS-PAGE

Rice flour (40 mg) was extracted in 0.5 mL of Tris-urea buffer at −20°C for 24 h. The extract was then centrifuged at 13,000 ×g for 30 min, after which the supernatant was collected for protein content measurement using the BCA assay with BSA as the standard. The extracted grain storage protein fractions were examined by SDS-PAGE using a Mini-PROTEIN® Tetra cell, 2-Gel system (Bio-Rad, USA), following the previously described methods [27]. Following electrophoresis, the gel was stained with Coomassie Blue R-250 and then destained with a destaining solution. The protein gel image was obtained with a Gel Doc system (Syngene GBOX-F3-E, USA). The intensity of each protein band of three biological replicates was evaluated using ImageJ version 1.52a (National Institutes of Health, USA).

## 3 Results

### 3.1 An overview of differential gene expression in RD6 and RD57 genotypes

**Fig. 1:**
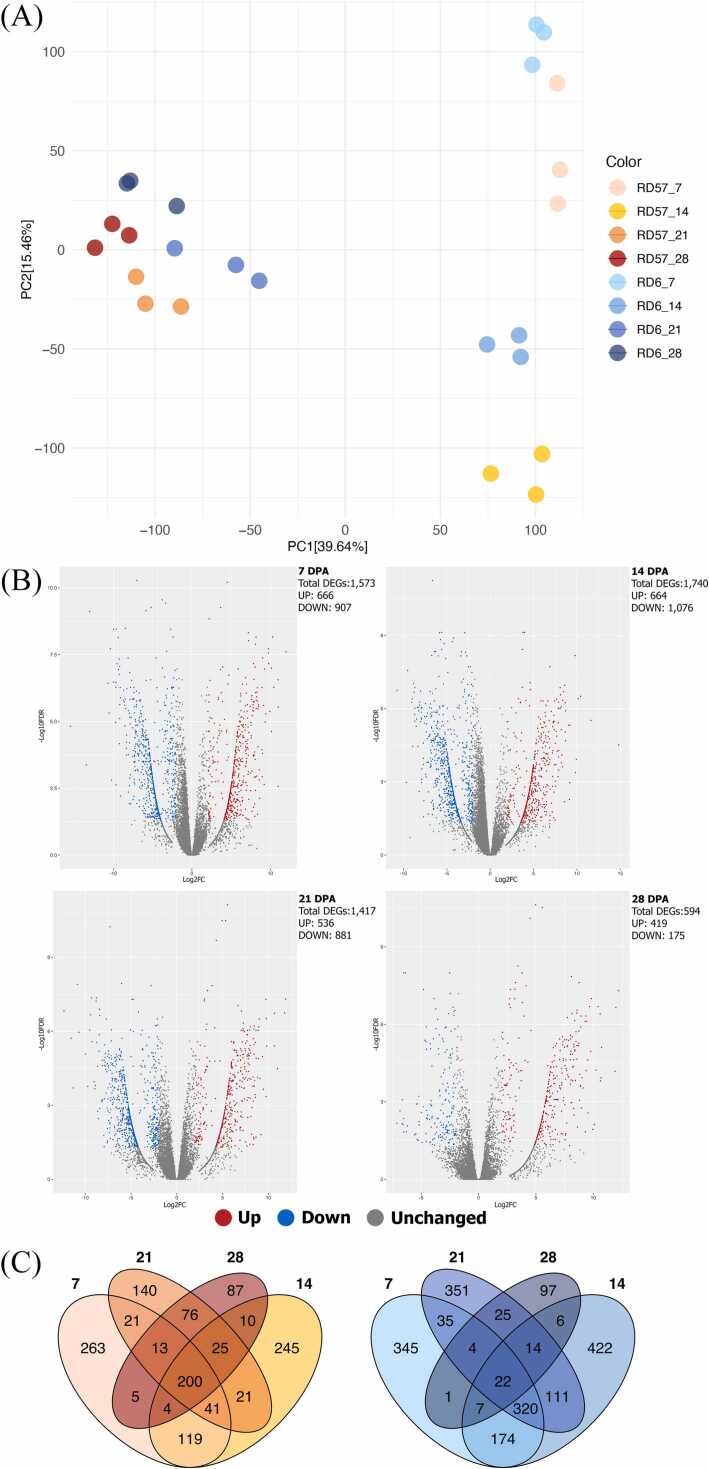
A comparative study of gene expression profiles in RD6 and RD57 rice genotypes. (A) PCA of gene expression levels from RD6 and RD57 genotypes. The first two principal components (PC1 and PC2) are illustrated with colors representing genotypes and time points. (B) Volcano plot of DEGs. Pairwise comparisons between RD57 and RD6 at each time point were conducted to identify DEGs. Log2FC represents the log2-fold change of gene expression, while the negative log10-transformed FDR or -Log10FDR is on the y-axis. Upregulated DEGs are highlighted in red, downregulated DEGs in blue and genes that are not significantly different are shown in gray. (C) Venn diagrams showing the overlap of upregulated DEGs (left) and downregulated DEGs (right) in RD57 compared to RD6 across the four DPAs.

The PCA revealed clear differences in gene expression between the young (7 and 14 DPA), and mature (21 and 28 DPA) grains for both genotypes (Fig. 1A). Grain transcriptional profiles at 7 and 14 DPA diverged, while those between 21 and 28 DPA were more similar (Fig. 1A). There were 5324 DEGs, with 1573, 1740, 1417, and 594 DEGs identified at 7, 14, 21, and 28 DPA, respectively (Fig. 1B). More genes were downregulated than upregulated when comparing RD57 to RD6 at 7, 14 and 21 DPA, in contrast to 28 DPA. Expression differences were highest at 14 DPA and lowest at 28 DPA (Fig. 1B).

Fig. 1 C illustrates genes that are uniquely or commonly up- or downregulated during grain-filling in RD57 compared with RD6 at each time point. Two hundred genes were upregulated in RD57 across time points higher than those downregulated (22 genes). Therefore, rice exhibits genotype-specific transcriptional responses at distinct developmental stages. The greatest numbers of up- and downregulated genes in RD57 were observed at 7 DPA (263 genes) and 14 DPA (422 genes), respectively. The number of commonly expressed genes was higher between consecutive time points; only a small subset, i.e., (six in total) was commonly expressed between the early (7 DPA) and late (28 DPA) stages. Genes underscoring storage product accumulation, including *AgpL2*, *AgpS2b*, *BEI*, *BEIIb*, *bZIP10-like*, *GluA-1.2*, *GluA-1.3*, *GluD-1*, *PHOL*, and *PUL*, were upregulated in RD6 at 28 DPA, while *GBSSI* was commonly upregulated at both 7 DPA and 21 DPA. In addition, *bZIP44-like* was commonly upregulated in RD6 at 7 DPA and 14 DPA, whereas *SNRK2.10* was downregulated in RD6 at 7 DPA.

### 3.2 Temporal dynamics of transcriptome profiles across seed development stages

**Fig. 2:**
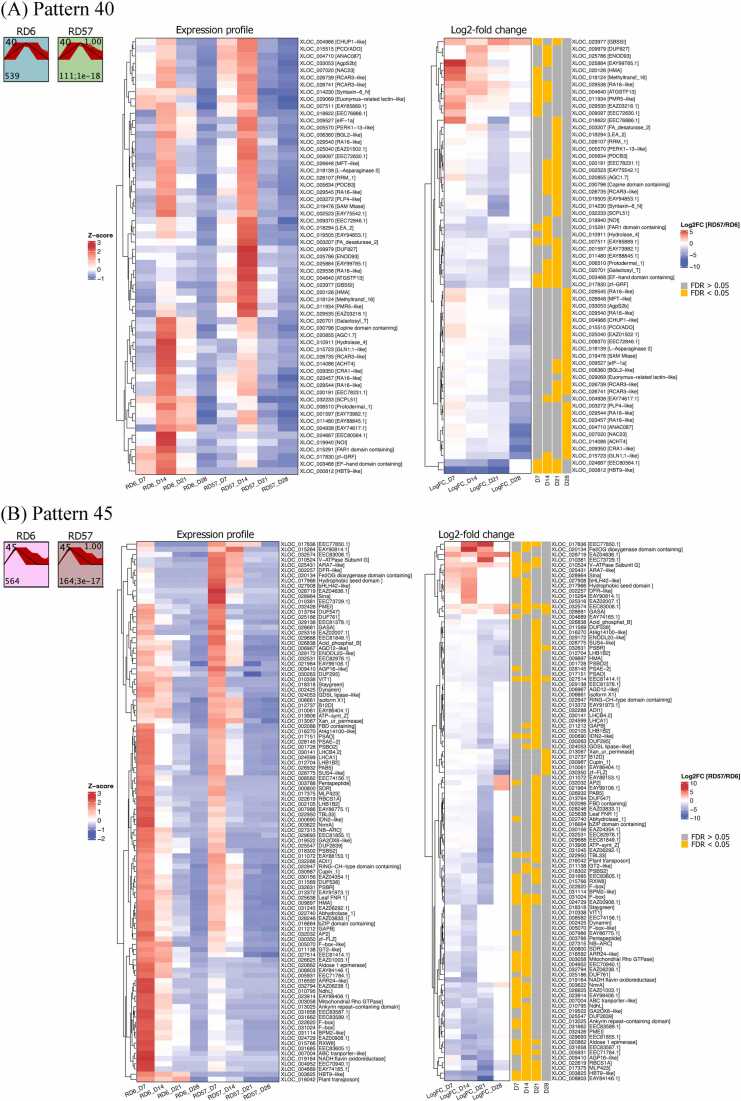
Significantly correlated temporal expression profile patterns between RD6 and RD57. (A) Pattern 40 and (B) Pattern 45. The temporal expression profiles of RD6 and RD57 are shown in the top row, with the pattern names in the top-left corner. The correlation coefficients, p-values, and the number of overlapping genes are displayed in the top-right and bottom-left corners of the RD57 box, respectively. Heatmaps of the overlapping genes between RD6 and RD57 in each pattern are shown. Each column represents a DPA, and each row is a gene. The left heatmap displays expression levels converted to z-scores for comparative analysis across time-points. Red indicates high expression levels, while blue represents low expression levels. The right heatmap shows Log2FCs in expression levels, with red and blue indicating upregulation and downregulation in RD57, respectively. Significant DEGs at each DPA are highlighted in yellow.

Significant temporal expression profiles were categorized into nine patterns for RD6 and ten patterns for RD57 (Fig. S4). Five patterns from RD6 and six patterns from RD57 showed active expression during early seed development (i.e., at 7 and 14 DPA). Eight pairs of significantly correlated profiles between RD6 and RD57 were observed (Fig. S5). The profile pairs of Pattern 40 and Pattern 45 were the primary focus (Fig. 2), as they contained genes involved in starch and storage protein biosynthesis.

Pattern 40 reflected active gene expression at 14 DPA (Fig. 2A), including starch biosynthetic genes, *AgpS2b* and *GBSSI,* in both RD6 and RD57. The expression profiles of *AgpL2*, *BEI*, *BEIIb*, *PHOL*, and *PUL* in RD6, as well as *GluA-1.2*, *GluA-1.3*, and *GluD-1* in RD57, also grouped into this pattern. Clustering of overlapping genes from both genotypes revealed genes such as *AtGSTF13* and *ENOD93* that were expressed significantly higher in RD57. Conversely, genes encoding EF-hand domain-containing, FAR1 domain-containing, zf-GRF, NOI, AGC1.7, Hydrolase_4, and Galactosyl_T were expressed at higher levels in RD6 (Fig. 2A).

The genes in pattern 45 were actively expressed at 7 DPA, followed by a gradual decrease in expression levels (Fig. 2B). Only the expression profiles of starch biosynthetic *BEI*, *BEIIb*, *PHOL* and *PUL* in RD 57 were assigned to this group. Among the common genes from RD6 and RD57 sharing this pattern, *Sina* and *V-ATPase Subunit G* were expressed at higher levels in RD57. In contrast, higher expression of genes encoding Ankyrin repeat-containing domain, F-box, NADH:flavin oxidoreductase, NdhL, Mitochondrial Rho GTPase, RXW8, AP2, Aldose 1-epimerase, NmrA, and TBL33 was observed in RD6 (Fig. 2B).

### 3.3 Co-expression networks of starch biosynthesis-related genes

From the co-expression analysis, the greatest number of significantly correlating pairs was observed at 21 DPA for Pattern 40 and at 7 DPA for Pattern 45, whereas the fewest pairs were detected at 28 DPA for both patterns (Table S5). Most correlated genes showed positive associations.

**Fig. 3:**
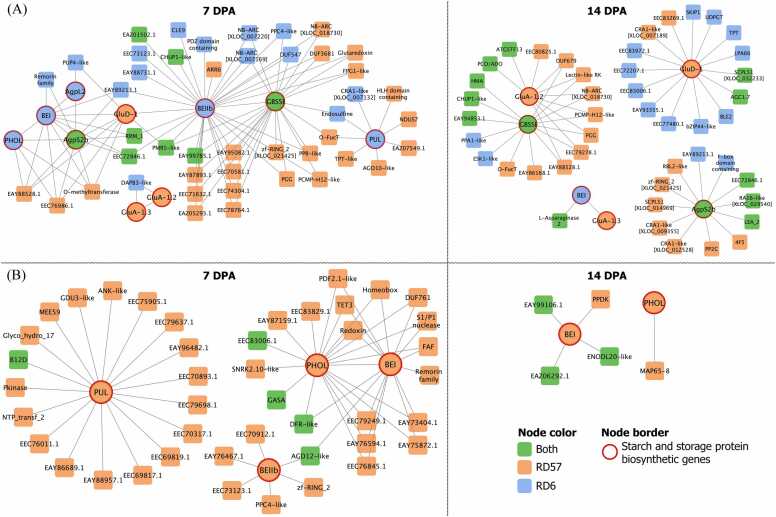
Co-expression networks of genes involved in starch biosynthesis. (A) Selected genes from Pattern 40 at 7 and 14 DPA. (B) Selected genes from Pattern 45 at 7 and 14 DPA. The color of the nodes corresponds to the rice genotypes, with green representing co-expressed genes common to both RD6 and RD57, blue for RD6-specific genes, and orange for RD57-specific genes. Starch and storage protein biosynthetic genes are represented as nodes with a red border.

Co-expression networks of starch biosynthetic genes were constructed for each DPA using only positive and significant correlations (*p* < 0.01) (Fig. 3 and Fig. S6). Pattern 40 showed more co-expressed genes than Pattern 45, with the largest networks at 21 DPA (204 pairs) for Pattern 40 and 7 DPA (58 pairs) for Pattern 45. Both patterns had the fewest pairs at 14 DPA (53 and 5 pairs, respectively). Several starch biosynthetic genes, including *AgpL2*, *AgpS2b*, *GBSSI*, *GluA-1.2*, *GluA-1.3*, and *GluD-1*, were co-expressed in Pattern 40, while *BEI*, *BEIIb*, *PHOL*, and *PUL* appeared in both patterns. Notably, *GBSSI* was only co-expressed with *BEIIb* at 7 DPA, and *BEI* and *GluA-1.3* formed a single pair of starch biosynthetic genes at 14 DPA of Pattern 40. For genes from Pattern 40, *AgpL2*, *GluA-1.2*, and *BEI* were co-expressed with the other starch biosynthetic genes, except *GBSSI*. *GluD-1* was not co-expressed with *GBSSI*, *PHOL*, or *PUL*, and *GluA-1.3* showed no associations with *AgpS2b*, *BEIIb*, *GBSSI*, *PHOL*, or *PUL* (Fig. 3A and Fig. S6A). For Pattern 45, *BEI* and *PHOL*, and *BEIIb* and *PUL* were co-expressed at 7 DPA and 28 DPA, respectively, while all four genes were co-expressed at 21 DPA (Fig. 3B and Fig. S6B).

A summary of the co-expression analysis is provided in Table S5, and detailed interpretations of these correlation pairs are discussed in the Discussion section.

### 3.4 Comparative expression patterns of starch biosynthesis and storage protein-related genes

**Fig. 4:**
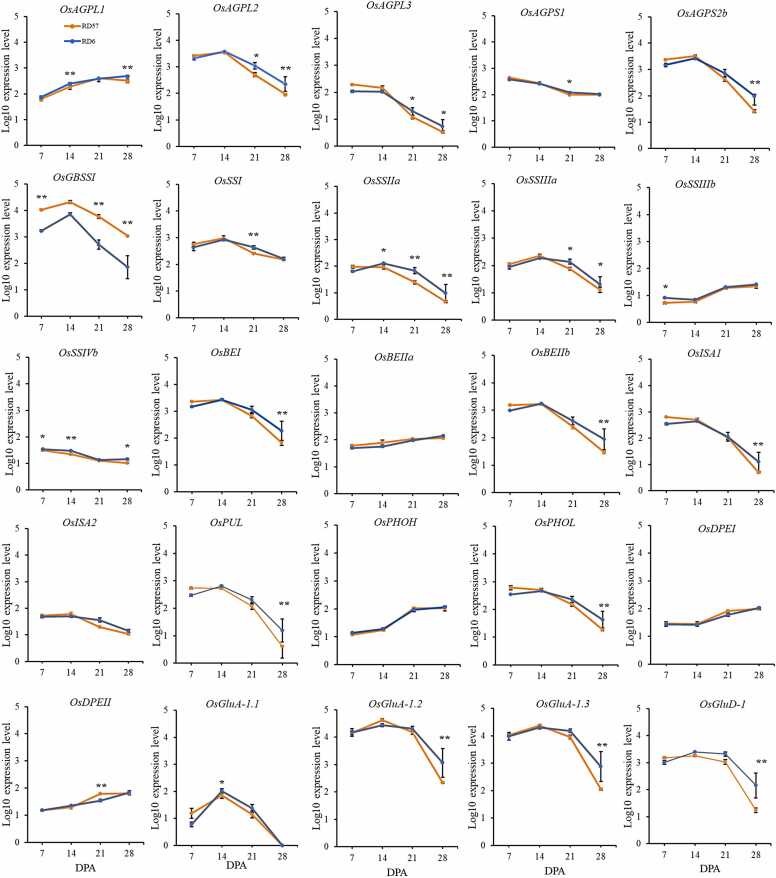
Variations in transcriptional expression of starch and main storage protein biosynthetic genes in developing seeds of RD6 and RD57. The x-axis shows the day of seed collection after anthesis (DPA). The y-axis displays log10 gene expression level based on RNA-Seq analysis. Data are represented as means ± SEM of three biological replicates. The * and ** symbols denote statistically significant differences in transcript levels between RD6 and RD57 at the same developmental stage with *p* ≤ 0.05 and *p* ≤ 0.01, respectively.

The expression patterns of starch and storage protein biosynthetic genes were observed and compared between RD6 and RD57 throughout caryopsis development (Fig. 4). *OsAGPL1, OsBEIIa, OsPHOH,* and *OsDPEII* mRNAs increased at 7 DPA and were maximal at 28 DPA in *waxy* RD6, whereas the expression levels of *OsBEIIa, OsSSIIIb, OsDPEI, and OsDPEII* increased at 7 DPA and reached a maxima at 28 DPA in the high-amylose RD57 rice (Fig. 4). The expression of *OsAGPL1 and OsPHOH* genes increased at 7 DPA, was highest at 21 DPA, and subsequently declined at 28 DPA in RD57 (Fig. 4). *OsAGPL1* expression was significantly lower in RD57 than in RD6 at the middle and late grain filling stage (14 and 28 DPA, respectively) (Fig. 4). At 21 DPA *OsDPEII* transcript was lower in RD6 than RD57, while at 7 DPA *OsSSIIIb* transcripts in RD6 was substantially higher than that in RD57 (Fig. 4). *OsSSIIIb* and *OsDPEI* transcripts in RD6 seeds declined from 7 to 14 DPA, then gradually increased to a maximum level at 28 DPA (Fig. 4).

Twelve genes initially increased expression at 7 DPA, reached a maximum level at 14 DPA, and gradually diminished to a minimum level at 28 DPA in both RD6 and RD57. These genes were *OsAGPL2, OsAGPS2b, OsGBSSI, OsSSI, OsSSIIIa, OsBEI, OsBEIIb, OsISA2, OsGluA-1.1, OsGluA-1.2, OsGluA-1.3,* and *OsGluD-1* (Fig. 4). Nonetheless, all of these genes except *OsISA2* were differentially expressed at certain development stages between the two rice cultivars (Fig. 4). *OsAGPL2* and *OsSSIIIa* mRNAs were higher in RD6 than in RD57 during the late grain filling stage (21 and 28 DPA) (Fig. 4), while for *OsGluA-1.1* it was at 14 DPA (Fig. 4). In contrast, *OsGBSSI* expression was 3- to 10-fold lower in RD6 rice grain compared to RD57 throughout grain filling (Fig. 4).

The expression of four genes, i.e., *OsSSIIa, OsISA1, OsPUL,* and *OsPHOL* peaked early and then decreased. In RD57, peak expression occurred at 7 DPA and decreased from 14 DPA onwards, whereas in RD6 these transcripts peaked later, at 14 DPA, and declined from 21 DPA (Fig. 4), suggesting important temporal regulation in their expression. *OsISA1, OsPUL,* and *OsPHOL* transcripts were also lower in RD57 than in RD6 at the late filling stage (28 DPA; Fig. 4). Interestingly, the expression level of the *OsSSIIa* gene in RD6 grain was significantly higher than that in RD57 from mid-to-late, i.e. 14–28 DPA (Fig. 4).

The expression of four genes including *OsAGPL3*, *OsAGPS1*, *OsSSIVb*, and *OsISA2* declined gradually from 7 to 28 DPA in both genotypes (Fig. 4). *OsAGPL3* mRNAs in RD6 were considerably higher than that in RD57 at 21 and 28 DPA, while the *OsAGPS1* mRNA in RD6 was only higher than that in RD57 at 21 DPA (Fig. 4). *OsSSIVb* expression was low, but in RD6, it was significantly higher than RD57 throughout grain filling at 7, 14 DPA, and 28 DPA filling stages (Fig. 4).

Overall, we demonstrated significant differences in the degree of expression of some starch biosynthetic genes and all storage protein genes in developing endosperms between the waxy RD6 and high-amylose RD57 cultivars. Nevertheless, no difference in their temporal pattern was observed between the two genotypes with different grain starch properties. This suggests that while the expression levels of starch and storage protein biosynthetic genes vary significantly between the two cultivars, their temporal regulation during the critical phase of grain filling was largely consistent.

### 3.5 Quantitative Real-time PCR (qRT-PCR) and enzymatic activity validation of grain starch and storage protein-related genes

**Fig. 5:**
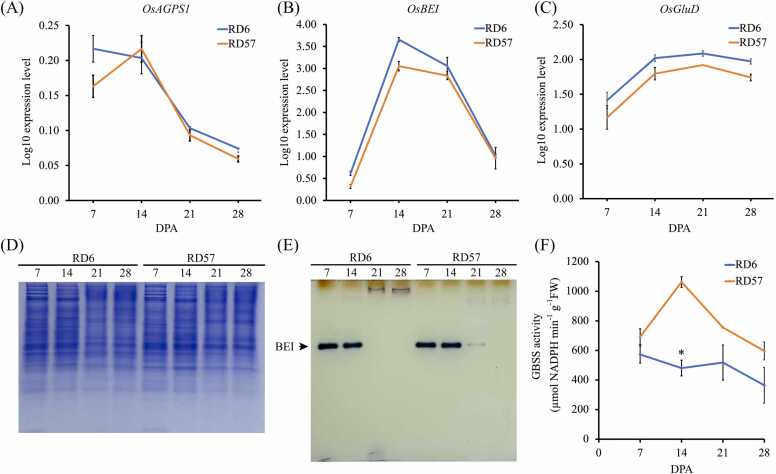
Validation of RNA Sequencing data with real-time quantitative PCR and enzymatic activity analyses. qRT-PCR analysis showing the log10 relative expression of **(A)***OsAGPS1,***(B)***OsBEI,* and **(C)***OsGluD* in developing seeds of RD6 and RD57 at 7, 14, 21, and 28 DPA. **(D)** Total proteins in developing endosperm of RD6 and RD57 at 7, 14, 21, and 28 DPA were separated by Native-PAGE and stained with Coomassie Brilliant Blue. **(E)** Activity staining of BEI and **(F)** GBSS activity in developing endosperm of RD6 and RD57. Data are represented as means ± SEM of three biological replicates. An asterisk (*) represents statistically significant differences in GBSS activity between RD6 and RD57 at the same developmental stage by independent Student’s *t*-test at *p* ≤ 0.05.

Transcripts of the differentially expressed starch and storage protein genes were compared to qRT-PCR (Fig. 5A-C), enzyme activity or protein staining (Figs. 5D and 5E), and activity assays (Fig. 5F). Results from our qRT-PCR analysis of *OsAGPS1*, *OsBEI*, and *OsGluD* (Fig. 5A-C) were similar to those from the RNA-seq data (Fig. 4), validating the gene expression data in this study. Native-PAGE/activity staining of BEI demonstrated that the protein level and enzymatic activity of the BEI isozyme (Fig. 5E) were consistent with the *OsBEI* expression pattern obtained from transcriptomic profiling (Fig. 4). On the Native-PAGE gel, the BEI-corresponding protein bands were most intense at 7- and 14 DPA in both RD6 and RD57 developing endosperms (Fig. 5E). The bands became faint at 21 DPA and were undetectable at 28 DPA in RD57 grain, whereas they were not observable at both 21 and 28 DPA in RD6 caryopses (Fig. 5E). This enzyme activity coincided with the RNA-seq (Fig. 4) and qRT-PCR (Fig. 5B) data indicating that the expression level of the *OsBEI* transcript in both genotypes increased from 7 DPA to reach its highest level at 14 DPA, and then rapidly declined to its lowest amount at 28 DPA (Fig. 4).

**Fig. 6:**
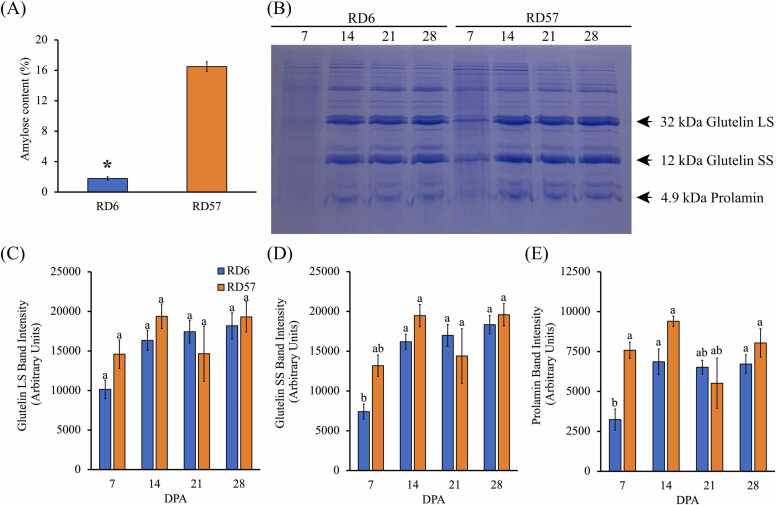
Variation in amylose content and storage protein composition of RD6 and RD57 endosperms. (A) Amylose content in the mature endosperm of RD6 and RD57. Values are mean ± SEM of three biological replicates. The vertical bar marked with an asterisk (*) indicates a statistically significant difference between RD6 and RD57 by independent Student’s *t*-test at *p* ≤ 0.05. (B) SDS-PAGE gel displaying glutelin LS, glutelin SS, and prolamin storage proteins from the RD6 and RD57 endosperm at 7, 14, 21, and 28 DPA. Densitometry plots displaying the protein band intensity of (C) glutelin LS, (D) glutelin SS, and (E) prolamin. The vertical bars represent the relative intensity of the target polypeptide bands. Values are means ± SEM of three biological replicates. Means with different superscripts are significantly different (*p* ≤ 0.05) by Tukey’s test.

The temporal pattern of *OsGBSSI* expression (Fig. 4) and GBSS enzyme activity (Fig. 5F) in RD57 throughout grain filling were similar, but these patterns differed in RD6 (Figs. 4 and 5F). In RD57, GBSS activity was high at 7 DPA, peaked at 14 DPA, and was strikingly lower at late grain filling stages (Fig. 5F). In RD6, GBSS activity fluctuated during grain filling and was markedly lower than that of the high-amylose RD57 cultivar across seed development (Fig. 5F). The GBSS activity, especially the GBSSI isoform, plays a major role in synthesizing amylose in the rice endosperm [12]. Consistently, the RD6 endosperm with a lower GBSS activity contained a noticeably lower amylose accumulation compared to RD57 (Figs. 5F and 6A).

### 3.6 Grain starch and storage protein composition

Waxy rice RD6 had 5-fold lower amylose than the high amylose rice RD57 (Fig. 6A). This agrees with the lower GBSS activity in RD6 endosperm compared to that of RD57 at 14 DPA (Fig. 5F). In addition, total crude proteins from RD6 and RD57 rice grains were extracted to examine the temporal expression patterns of the glutelin large (LS) and small subunit (SS), and the prolamin storage proteins. The protein bands of the two major grain storage proteins, 4.9 kDa prolamin and glutelin (32 kDa glutelin LS and 12 kDa glutelin SS), were visible on the discontinuous polyacrylamide gel (Fig. 6B). Densitometry plots of the bands of the three proteins indicated that they were barely detectable at 7 DPA and then increased and remained constant after 14 DPA in both genotypes (Fig. 6C-E). This is consistent with the temporal expression patterns of various glutelin mRNAs in RD6 and RD57 caryopses (Fig. 4). *OsGluA-1.1*, *OsGluA-1.2*, *OsGluA-1.3*, and *OsGluD-1* were activated at 7 DPA, reached a maximum level at 14 DPA, and gradually declined from 21 DPA (Fig. 4).

### 3.7 *In silico* TF binding motif prediction

**Fig. 7:**
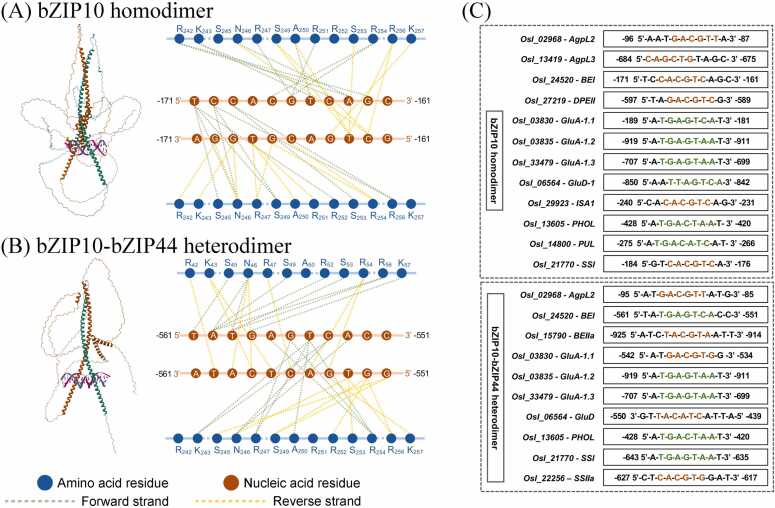
TF–DNA motif predictions of bZIP10 and bZIP44 with starch and storage protein biosynthetic gene promoters. (A–B) Predicted DNA-binding models of the bZIP10 homodimer and bZIP10–bZIP44 heterodimer complexed with the *BEI* promoter. The diagrams show interactions between protein secondary structures and double-stranded DNA. Blue and orange circles represent amino acid residues and nucleic acid motifs, respectively. Bonding interactions are color-coded: green for the forward and yellow for the reverse strand. (C) Predicted promoter regions containing bZIP10 homodimer and bZIP10–bZIP44 heterodimer motifs upstream of 12 and 10 starch biosynthetic genes, respectively. Conserved motifs are highlighted in orange for the G-box (5′-(G/C)ACGTG-3’) and C-box (5′-(G/C)ACGTC-3’), and in green for the A-box (5′- T(G/T)A(G/C)T(C/A)(A/C)-3′).

We further performed *in silico* predictions of bZIP binding motifs among starch biosynthetic genes. The bZIP10 protein formed a homodimer, whereas bZIP44 formed a heterodimer with bZIP10, consistent with previous studies [61], [62]. Structural predictions of the bZIP10 homodimer and bZIP10–bZIP44 heterodimer revealed leucine-zipper–mediated DNA contacts typical of bZIP TFs. Both complexes showed the canonical N-(x₇)-R binding signature [55]. The bZIP10 homodimer was predicted to bind the *BEI* promoter at –171 to –161, recognizing a C-box–like motif (5′-CACGTC-3′) (Fig. 7A). The bZIP10–bZIP44 heterodimer would bind the –561 to –551 region, interacting with an A-box–like motif (5′-TGACTCA-3′) (Fig. 7B). In total, 19 and 16 TF binding sites were identified for the homo- and heterodimers, targeting 12 and 10 starch and storage protein genes, respectively (Table S7). These results suggest that bZIP10 and bZIP44 recognize conserved bZIP cis-elements, including G-, C-, and A-box motifs [63], [64], [65] (Fig. 7C), and may regulate starch and storage protein biosynthetic gene expression. As targets for improving indica rice starch, further study of these TF interactions with starch and protein genes should be examined via ChIP-seq, transgenesis, or gene editing approaches.

## 4 Discussion

RD6 and RD57 indica rice starch exhibited distinct compositions and molecular structures, associated with differences in their physicochemical and functional properties [9], thereby influencing the nutritional value, ECQ, and health benefits of cooked rice grains. Developing new Thai commercial rice varieties with desired grain traits requires an in-depth understanding of the regulatory mechanisms controlling both starch and storage protein accumulation at both the transcriptional and post-translational levels. We thus investigated the gene expression patterns and enzymatic activities of major starch and storage protein biosynthetic enzymes in developing endosperms of these two indica rice genotypes. The results showed that most storage composition-related genes in both RD6 and RD57 were expressed at high levels from 7 to 21 DPA (Fig. 4), which likely contributed to notable increases in starch accumulation and grain weight throughout development [11]. Although there were small variations in the temporal expression patterns of genes associated with seed starch biosynthesis between waxy RD6 and high-amylose RD57 rice, significant differences in their expression levels were only observed at distinct grain-filling stages (Fig. 4).

### 4.1 Temporal expression of starch biosynthesis genes and their putative regulators

#### 4.1.1 ADPglucose biosynthesis

The large (AGPL) and small (AGPS) subunits of *AGPase* showed distinct expression patterns. *AGPL1* at 14 and 28 DPA and *AGPL2* and *AGPL3* at 21 and 28 DPA were higher in RD6 than RD57 (Fig. 4). In addition, *AGPS1* and *AGPS2b* genes were expressed higher in RD6 at 21 and 28 DPA, respectively (Fig. 4). Reduced GBSSI activity and amylose synthesis in RD6 (Fig. 5F and Fig. 6A) drastically alter cellular sugars [66] and may explain the generally higher expression of most AGPase subunits in RD6 due to their sensitivity to transcriptional sugar signaling [66]. Interestingly, the temporal expression pattern of *AGPL2* and *AGPS2b* the two cytosolic isoforms that account for more than 80 % AGPase activity in rice endosperm [12], [22], [67] were similar in both genotypes, with high expression from 7 to 21 DPA (Fig. 4). *AGPL2* and *AGPS2b* expression also matched the expression patterns and levels of genes encoding GBSS and SS throughout grain development of RD6 and RD57 (Fig. 4).

#### 4.1.2 Putative regulators of starch precursor biosynthesis

Co-expression gene networks (Fig. 3 and Fig. S6) identified putative regulation of the starch genes *AGPL2*, *AGPS2b*, *BEIIb*, and *PUL* in RD6 and RD57 by the ABA receptor 3-like (RCAR3-like) at 28 DPA (Fig. S6A). The regulatory components of ABA receptors (RCARs), particularly the pyrabactin resistance 1-like (PYR/PYL) family of receptors, mediate ABA functions in cereal grains [68], [69]. ABA regulates starch biosynthesis in cereal grains through their action on the expression of *AGPase*, *SSI*, *SSIIIa*, and *ISA1* [22], [70].

#### 4.1.3 Amylopectin and amylose biosynthesis

The expression of starch biosynthetic genes SS (isoforms *IIIb*, *I, IVb*, *IIa*, and *IIIa*)*, BE (*isoforms *I* and *IIb*), *ISA1*, *PUL*, and *PHOL* were higher in RD6 compared with RD57, depending on the filling stage examined (Fig. 4). *SSIIIb* and *SSI* expression were higher at 7 and 21 DPA, respectively, *SSIVb*, *SSIIa*, and *SSIIIa* at early, middle, and late grain filling stages respectively, and *BEI*, *BEIIb*, *ISA1*, *PUL*, and *PHOL* expression was higher at the late grain filling stage (28 DPA) of RD6 vs. RD57 (Fig. 4).

SS, BE, and DBE play a major role in synthesizing the amylopectin backbone [67], [71], [72], and this glucan is five-fold higher in RD6 compared to the non-glutinous RD57 rice [9]. The SS isoforms *I*, *IIa,* and *IIIa*, the BE isoforms *I* and *IIb*, and the DBEs *ISA1* and *PUL* were expressed at higher levels in RD6 than in RD57 rice during the grain filling (Fig. 4). Based on the chain length preference of these SS and BE isoforms [67], [71], [73], [74], our differential gene expression data in RD6 vs. RD57 were consistent with the amylopectin fine structure of their starches [9]. Accordingly, the RD6 starches had significantly higher swelling power, gelatinization temperature range, and gelatinization enthalpy and considerably lower solubility, peak gelatinization temperature, retrogradation temperatures, pasting temperature, setback, and resistant starch (RS) content than those of the high-amylose RD57 starch [9]. These RD6 grains may require more time and energy to cook, but provide a soft texture for several hours after cooking [9], [25]. On the other hand, the RD57 grains may give a hard texture and become less adhesive upon cooking, but its cooked grains may be healthier due to the higher content of RS, which functions like fiber [9], [25], [75].

#### 4.1.4 Transcriptional and post-transcriptional regulation of starch polymer biosynthesis

*BEI* gene expression (Fig. 4) was consistent with the BEI activity patterns displayed on the activity staining Native-PAGE (Fig. 5E). BEI activity was high in both cultivars at 7 and 14 DPA (Fig. 5E). Although the BEI isozyme was visible (Fig. 5D), its activity could not be detected at 21 and 28 DPA (Fig. 5E). BEI activity was elevated during the early and middle stages of grain filling [12], [71], [73] and is essential for the formation of long glucan chains that provide a starter for the formation of a new amylopectin cluster [12], [71], [76]. BEI may facilitate the functions of SSI, SSIIa, SSIIIa, and BEIIb to extend and form branch chains of the amylopectin cluster structure, and concertedly construct starch granules during the early and middle developmental stages [67], [71], [77]. Accordingly, our results showed that BEI expression pattern was comparable to those of SSI, SSIIa, SSIIIa, and BEIIb across the grain filling stage (Fig. 4).

Network analysis revealed that the *bZIP10-like* gene was co-expressed with several genes related to starch and storage protein biosynthesis pathways in RD6 and RD57 rice seeds at 21 DPA (Fig. S6A). The bZIP family of TFs in Japonica rice [22], [78], specifically *OsbZIP58*, were identified as key transcriptional activators of both starch synthesis-related genes (e.g., *OsAGPL3*, *Wx*, *OsSSIIa*, *OsBEI*, *OsBEIIb*, and *OsISA2*) and storage protein genes (e.g., *OsGluA1*, *OsGluA2*, *OsGluA3*, *OsGluB1*, and *OsGluD1*) [79]. It is interesting to see whether the identified bZIP10-like protein is also involved in the regulatory networks that control the production of grain starch and storage proteins in indica rice.

The two types of starch DBEs, ISA and PUL [1], [12], trim excess branches in amylopectin, contributing to the formation of a more crystalline and organized starch structure [12], [71]. Mutations in *ISA1* may lead to the accumulation of phytoglycogen [80] and alterations in the distribution of amylopectin chain length and branching patterns [81], which directly impact rice ECQ (i.e., gelatinization behavior, texture, and digestibility) [71], [80]. In this study, the glutinous high-amylopectin RD6 rice grain at 28 DPA had markedly higher levels of *ISA1* and *PUL* transcripts relative to the non-glutinous RD57 rice grain at the same developmental stage (Fig. 4), consistent with their differences in the proportion and fine structure of amylopectin [9]. The coordinated action of DBEs and starch PHO enzymes are fundamental to maintaining starch homeostasis in cereal grains [82].

Starch phosphorylase L (PHOL), localized to the plastid (Pho1: L type), catalyzes the reversible phosphorolysis of α-1,4-glucosidic linkages, releasing glucose-1-phosphate [82], [83]. PHOL is important for both the synthesis and degradation of starch, depending on the plant's developmental stage and energy requirements [82], [83]. Our co-expression networks demonstrated that the *SnRK2.10–like* gene was co-expressed with the *PHOL* at 7 DPA (Fig. 3B). While there is no direct evidence linking SnRK2.10-like to PHOL, it is noteworthy that the other member of the SnRK family, particularly SNF1-related protein kinase 1 (SnRK1), is associated with the regulation of starch biosynthesis enzymes [84]. Overexpression of *TaSnRK1α* led to increased starch content via enhanced activity of AGPase [85]. Thus, in addition to SnRK1, SnRK2.10-like protein may influence starch metabolism through modulation of specific enzymes.

*BEI* expression aligned with the expression patterns of *GBSSI* (Fig. 4) and GBSSI activity (Fig. 5F) throughout grain development of the non-glutinous RD57 rice. BEI and GBSSI may interact during the synthesis of amylose linear chains of the developing RD57 rice endosperm [67]. GBSSI catalyzes the linear chain elongation of amylose and amylopectin, while SBEI preferentially transfers shorter glucan chains to create a few branches via an α-1,6-glucan linkage [12], [67]. Similarly, the expression pattern of *BEI* also aligned with that of *GBSSI* in glutinous RD6 grain (Fig. 4).

The glutinous RD6 rice has the mutant *GBSSI* (waxy; *Wx*) gene [12], [86] and lower *GBSSI* transcript (Fig. 4) and enzymatic activity (Fig. 5F) throughout endosperm development, leading to 5-fold less amylose than RD57 starch (Fig. 6A). RD6 starch has a lower pasting temperature, final viscosity, and setback values compared to the RD57 starch [9], which gives it a stickier and softer texture and shorter cooking time compared to RD57 rice grain [9], [25]. Nonetheless, *GBSSI* mRNA accumulation was not consistent with its enzymatic activity, particularly at 7 DPA (Fig. 4 and Fig. 5F). Differences between *GBSSI* mRNA and enzyme activity can stem from defects in protein targeting/granule-binding, protein stability, or post‑translational modifications [87]. In rice endosperm GBSSI is normally localized in the amyloplast and tightly bound to the starch granule, and proper targeting/anchoring—mediated by starch‑binding adaptor proteins such as PTST and its rice homologs (OsGBP/PTST‑like)—is essential for enzyme stability and function [66], [87]. The waxy (*Wx*) mutation in RD6 may disrupt these adaptor interactions, causing accumulation of soluble, non‑granule‑bound GBSSI that is unstable and rapidly degraded proteolytically [66], [87]. Thus, despite the normal or elevated levels of *Wx* transcripts, GBSSI enzyme activity is markedly reduced due to post-translational destabilization of the mislocalized protein [66], [87].

Understanding the underlying mechanisms regulating *GBSSI* expression would allow the improvement of starch quality in indica rice cultivars. Here, gene networks highlighted co-expression between the *PPA1-like* gene encoding pyrophosphatase (PPase), and *GBSSI* in the endosperms of both RD6 and RD57 at 14 DPA (Fig. 3A). The pyrophosphatase enzyme may indirectly regulate the activity of the GBSSI enzyme by modifying the inorganic pyrophosphate (PPi.) levels in amyloplast [12], [88]. The accumulation of PPi can inhibit AGPase activity, [12], [88] and PPases plays a critical role [12], [88] by hydrolyzing PPi into two inorganic phosphate molecules, effectively driving the AGPase reaction thermodynamically forward [12], [88]. The co-expression of *PPA1-like* with *GBSSI* could ensure successful starch synthesis, especially in RD57 rice caryopses with high amylose production. We also identified genes encoding DUF630 and DUF632 domains that were co-expressed with *GBSSI* in the developing endosperms of both rice genotypes at the late filling stage (28 DPA) (Fig. S6A). This is important because a recent study identified a rice protein FGW1 with DUF630 and 632 domains, which positively regulates AGPase, SS, and sucrose synthase (SuSy) activity, grain filling, grain width, and grain weight [89], opening the possibility that DUF630- and 632-containing proteins are important determinants for the development and quality of rice grains.

### 4.2 Temporal expression of storage glutelin biosynthesis genes and their putative regulators

The glutelin genes marginally increased from 7 to 14 DPA and gradually declined at 21 DPA onwards (Fig. 4), coinciding with the temporal accumulation of 32 kDa glutelin LS and 12 kDa glutelin SS in RD6 and RD57 (Fig. 6C and Fig. 6D). Although *OsGluA-1.1, OsGluA-1.2, OsGluA-1.3,* and *OsGluD-1* transcripts were higher in RD6 than in RD57 at 14 DPA and at 28 DPA, respectively (Fig. 4), the accumulation of glutelin LS, glutelin SS, and prolamin proteins were not (Fig. 6C-E). This discrepancy suggests the involvement of regulatory controls downstream of transcription [90]. Contributing mechanisms may include mRNA localization and translational efficiency of storage protein genes [90]. In rice endosperm, glutelins and prolamins mRNAs are localized to distinct subdomains of the endoplasmic reticulum [90]. The correct localization of glutelin and prolamin mRNAs is determined by RNA-binding proteins (RBPs); mutations in these RBPs causes mislocalization of the transcripts and disruption of protein accumulation and deposition [90]. Post-translational modifications also shape final protein levels [91]. Proteolytic cleavage, glycosylation, and disulfide‑bond formation are required for storage protein folding, stability, and function, and limited activity of modifying enzymes or chaperones can constrain maturation [91]. For glutelins, inefficient cleavage into acidic and basic subunits can lead to precursor accumulation or degradation, lowering mature glutelin despite high transcript abundance [91]. Glutelin make up 60–80 % of rice grain proteins [92] and determine the nutritional value and quality of rice grain, by affecting the pasting and textural properties of cooked rice, starch digestibility, overall health benefits, and industrial applications [22], [92].

Network analysis revealed the co-expression between *DAPB3-like* and *GluA-1.2* and *GluA-1.3* genes in the two rice endosperms at 7 DPA (Fig. 3A). The *DAPB3-like* gene presumably encodes a dihydrodipicolinate reductase (DHPR), a key enzyme in the lysine biosynthetic pathway. It catalyzes the reduction of dihydrodipicolinate to tetrahydrodipicolinate, a crucial step in the production of lysine, an essential amino acid often limited in cereal grains [93], [94], [95]. *GluA-1.2* and *GluA-1.3* genes encode glutelins that are rich in glutamine and proline but typically low in lysine [92], [96]. The activity of DHPR could influence lysine availability, which is a limiting amino acid in glutelin. Previous studies have demonstrated that enhancing DHPR activity can potentially increase lysine content, thereby improving the nutritional quality of glutelin proteins in cereal grains [93], [94], [95].

In cereal grains, misfolded or improperly assembled glutelin polypeptides are ubiquitinated by ubiquitin ligases and subsequently degraded via the ubiquitin–proteasome system, thereby preventing the accumulation of defective proteins and preserving cellular homeostasis and optimal seed protein composition [97]. We identified co-expression between the *ASK4-like* gene and the *GluA-1.3* and *GluD-1* genes in RD6 and RD57 at 21 DPA (Fig. S6A). The *ASK4-like* gene encodes SKP1-like protein 4, which is related to ubiquitination and subsequent proteasomal degradation of target proteins [98].

The expression of storage protein genes is tightly regulated during seed development to ensure proper nutrient composition [99]. Histone modifications, particularly histone monoubiquitination, was previously highlighted to regulate DNA replication, repair, and transcription of various genes in wheat [100]. In this study, we observed that the *GluA-1.3* and *GluD-1* genes were co-expressed with the *HTB9-like* gene encoding a histone 2B (H2B) protein in both rice genotypes at 21 DPA (Fig. S6A). These modifications potentially influence the expression of genes encoding storage proteins such as glutelin [99].

The *bZIP44-like* gene was co-expressed with the *GluD-1* gene in grains of both cultivars at 14 DPA (Fig. 3A). In *Arabidopsis thaliana* bZIP44 influences responses to iron deficiency stress through cooperation with other TFs like MYB10 and MYB72 [101]. However, given the functional diversity within the bZIP family, bZIP44-like proteins may participate in the complex regulatory networks governing the biosynthesis of glutelin storage proteins in RD6 and RD57 rice grains. Furthermore, the absence of globulin negatively impacted the transcriptional regulation of glutelin genes during seed development [102]. Rice mutants with suppressed globulin expression (*Glb*-RNAi lines) showed a comprehensive reduction in the expression of various glutelin genes (i.e., *GluA-1*, *GluB-1a*, *GluC-1*, and *GluD-1*) [102]. Consistent with this, our results revealed that the *CRA1-like* gene encoding globulin type B2 was co-expressed with *GluA-1.2*, *GluA-1.3*, and *GluD-1* in the RD 6 and RD57 endosperms at 28 DPA (Fig. S6A).

### 4.3 Comparative expression of storage composition biosynthesis genes and their impacts on grain functionality

**Fig. 8:**
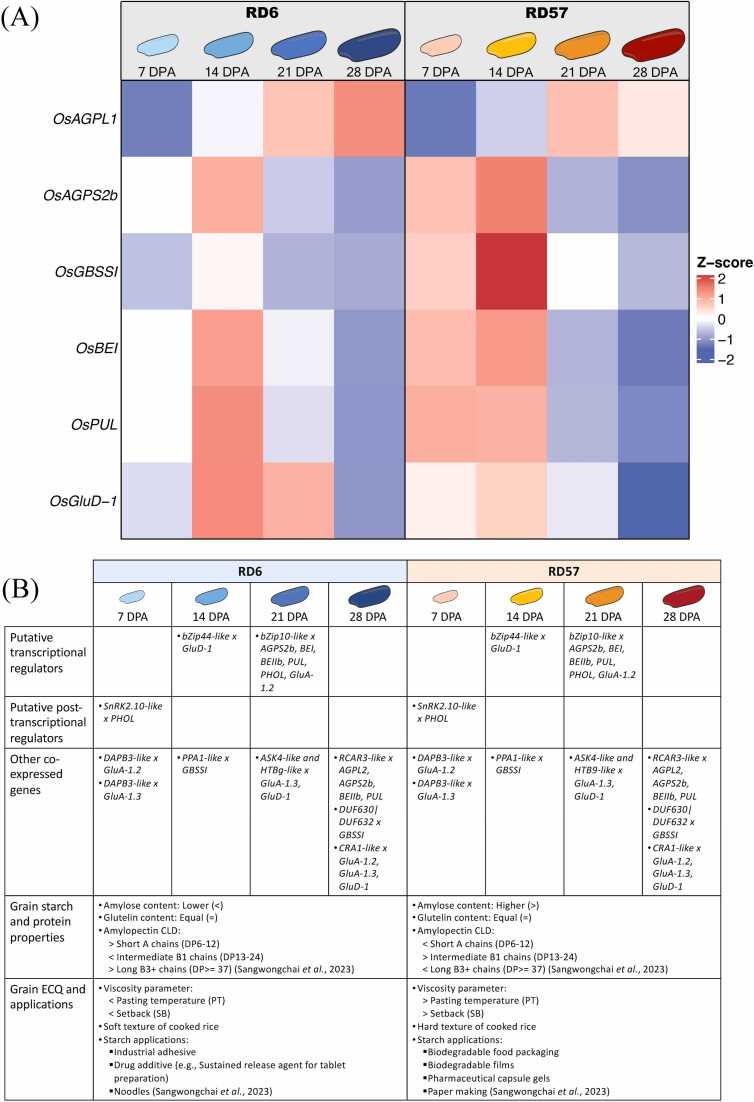
Comparison of the expression levels of starch and storage protein biosynthetic genes during grain filling and its influences on grain chemical compositions and starch functionality between waxy rice RD6 and high-amylose rice RD57. (A) Heat maps displayed expression levels of the starch and storage glutelin biosynthesis genes during grain filling of the RD6 and RD57 rice cultivars. (B) Putative molecular regulators during grain filling and comparative grain chemical compositions, starch molecular structures, starch physicochemical properties, and industrial applications of the two Thai rice varieties RD6 and RD57. A summary of correlation values is provided in Table S6.

Our comprehensive results are integrated and illustrated in Fig. 8. Most starch biosynthetic genes (i.e., *OsAGPS2b*, *OsGBSSI*, *OsBEI*, and *OsPUL*) and storage protein biosynthetic gene (i.e., *OsGluD-1*) in waxy rice RD6 exhibited weaker expression than in the high-amylose rice RD57 at 7 DPA (Fig. 8A). On the other hand, the levels of *AGPL1*, *AGPS2b*, *BEI*, *PUL*, and *GluD-1* mRNAs were significantly higher in RD6 compared to RD57 at the late grain filling stage (21- and 28 DPA; Fig. 8A). Interestingly, the expression level of *GBSSI* was significantly higher in RD57 than in RD6 across grain filling (Figs. 4 and 8A). GBSSI activity was presumably supported by the PPA1-like gene product, as suggested by their co-expression pattern in the developing endosperm of RD57 (Fig. 3A). It was also suggested that amylose synthesis in the RD57 developing grain primarily occurs between 7 and 14 DPA (Fig. 8A), which aligned with the period of active *PPA1-like* gene expression. Consistently, a 121 % higher activity of GBSSI was observed in RD57 endosperm compared to RD6 grain at the critical period (14 DPA) (Fig. 5F). This difference in amylose content could contribute to the higher pasting temperature and setback values of RD57 starch relative to waxy RD6 starch (Fig. 8B) [9]. Therefore, RD57 caryopses may require more energy to cook and provide a harder texture of cooked rice (Fig. 8B) [9], [25]. A large amount of long, linear amylose is also suitable for certain applications such as secondary biodegradable food packaging, pharmaceutical capsule gels, and paper making (Fig. 8B) [22], [103].

*OsAGPS2b and OsBEI* genes were expressed at significantly higher levels in RD6 grain than in RD57 grain at 21 DPA and 28 DPA (Fig. 8A). They may remain in the aggregation state of the soluble starch synthase enzymes and BEI in RD6 grain during the late grain-filling stage [104]. BEI, along with other starch biosynthetic enzymes, may forms complexes during this period to facilitate efficient amylopectin synthesis in RD6 grain [104]. *OsPUL* was expressed at significantly higher levels in the high amylopectin-RD6 than in the RD57 endosperm during the mid-to-late grain development (Fig. 8A), presumably to maintain amylopectin structure and starch crystallinity during this stage [9], [105]. Interestingly, we also found that in both cultivars, *BEI* was co-expressed with the TF *bZIP10-like* gene at 21 DPA, while the *PUL* was co-expressed with TF *bZIP10-like* and *RCAR3-like* genes at 21 DPA and 28 DPA, respectively (Fig. S6A and Fig. 8B). This suggested that the variations in *BEI* and *PUL* transcript levels between the two rice cultivars may be driven by the differential regulation of bZIP10-like TF and the *RCAR3*-like gene products. In addition to BEI roles in the formation of amylopectin branched structure [12], [67], [71], it also transfers longer chains that link multiple clusters of amylopectin in the amorphous lamella [106], [107]. Thus, its activity can increase the proportion of amylopectin long chains with a degree of polymerization (DP) ≥ 37 [106], [107]. In accordance with this, we previously reported that RD6 endosperm starch contained larger proportions of amylopectin long B_3+_ chains (DP ≥ 37) and amylopectin short A chains (DP 6–12) than RD57 grain (Fig. 8) [9]. The RD6 starch with a larger amount of amylopectin and a higher proportion of amylopectin long B*_3 +_* chains may comprise a higher -OH content, making it a more suitable raw materials for industrial use as an adhesive [22] and may be used as a sustained-release agent for tablet manufacture in pharmaceutical and supplement industries (Fig. 8B) [108]. In addition to *BEI* and *PUL*, *bZIP10-like* also co-expressed with *AGPS2b*, *BEIIb*, and *PHOL* at 21 DPA in both genotypes (Fig. 8).

Our *in silico* predictions of bZIP binding motifs among starch and storage protein biosynthetic genes further identified the bZIP10 homodimer putative binding to the *BEI* promoter, recognizing a C-box–like motif (5′-CACGTC-3′) (Fig. 7A). The bZIP10–bZIP44 heterodimer would also interact with an A-box–like motif (5′-TGACTCA-3′) (Fig. 7B). Several TF binding sites were also identified for the homo- and heterodimers, targeting 12 and 10 starch and storage protein genes, respectively (Fig. 7C). Therefore, we recommend functionally characterizing these TFs using ChIP-seq, transgenesis, or gene-editing approaches to further identify targets for rice breeding programs.

## 5 Conclusions

Expression of starch and storage protein biosynthesis genes, starch‑biosynthetic enzyme activities, and grain chemical compositions differed significantly between waxy RD6 and the high‑amylose RD57. Temporally, most starch and glutelin biosynthetic genes were higher in RD6 at late developmental stages, suggesting extended grain filling in this cultivar. *GBSSI* transcripts and GBSS activity were consistently higher in RD57 across development, while *SSIIa* expression was higher at mid‑to‑late developmental stages in RD6 endosperm. Co‑expression analysis implicated putative TFs (i.e., bZIP10-like and bZIP44-like), putative post-transcriptional regulator (i.e., SnRK2.10-like), and several co‑expressed genes (*DAPB3‑like*, *PPA1‑like*, *ASK4‑like*, *HTB9‑like*, *RCAR3‑like*, *CRA1-like*) with putative functions in storage biomolecule biosynthesis. TF–DNA predictions also suggested potential binding of bZIP10 homodimer and bZIP10-bZIP44 heterodimer with several starch and storage protein genes. Functional characterization of these candidate genes would ensure their implications for molecular breeding and gene editing. This transcriptional profiling of Thai indica rice—an understudied ecotype—offers a comprehensive view of grain storage molecule biosynthesis and can guide breeders and geneticists in improving its eating quality and health benefits.

## Abbreviations

ADP glucose pyrophosphorylase: AGPase
Basic leucine zipper: bZIP
Days post-anthesis: DPA
Debranching enzyme: DBE
Degree of polymerization: DP
Differentially expressed gene: DEG
Disproportionating enzyme: DPE
Glutelin type-A1: GluA-1
Glutelin type-D1: GluD-1
Granule-bound starch synthases: GBSS
Isoamylase 1: ISA1
Pasting temperature: PT
Phosphorylase: PHO
Phosphorylase L: PHOL
Pullulanase: PUL
Real-time quantitative PCR: RT-qPCR
RNA Sequencing: RNA-Seq
Setback: SB
Soluble starch synthase: SS
Starch branching enzyme: SBE
Transcription factor: TF

## Funding

This work was supported by the Fundamental Fund of Khon Kaen University, which has received funding support from the National Science Research and Innovation Fund (NRSF). The funder has no role in study design; in the collection, analysis and interpretation of data; in the writing of the report; and in the decision to submit the article for publication.

## CRediT authorship contribution statement

**Wichian Sangwongchai:** Writing – original draft, Visualization, Methodology, Investigation, Formal analysis, Data curation. **Kwanjeera Wanichthanarak:** Writing – original draft, Visualization, Software, Methodology, Investigation, Formal analysis, Data curation. **Sakda Khoomrung:** Writing – review & editing, Supervision, Resources. **Maysaya Thitisaksakul:** Writing – review & editing, Resources, Project administration, Methodology, Investigation, Funding acquisition, Formal analysis, Conceptualization. **Nutcha Sa-ingthong:** Methodology, Investigation, Formal analysis. **Diane M. Beckles:** Writing – review & editing, Supervision, Methodology. **Chamaiporn Champasri:** Visualization, Methodology, Investigation, Formal analysis. **Ammarin In-on:** Writing – review & editing, Visualization, Software, Methodology, Investigation, Formal analysis. **Supidcha Natee:** Visualization, Methodology, Investigation, Formal analysis, Data curation.

## Declaration of Competing Interest

The authors declare that they have no known competing financial interests or personal relationships that could have appeared to influence the work reported in this paper.

## Data Availability

The datasets in this study are available at GEO database (https://www.ncbi.nlm.nih.gov/geo/), GEO accession: GSE294589.

## Footnotes

Appendix A Supplementary data associated with this article can be found in the online version at doi:10.1016/j.csbj.2025.11.011.
