# Supplementary material for: Unveiling distinct storage composition and starch properties in developing indica rice grains via transcriptional profiling and enzymatic activity analysis
Source: Comput Struct Biotechnol J. 2025 Nov 7;27:4898–914. doi: 10.1016/j.csbj.2025.11.011 (PMC12657317; doi:10.1016/j.csbj.2025.11.011)
Supplement: Supplementary file 1 — Supplementary material [file mmc1.docx]

**Unveiling distinct chemical composition and starch properties in developing indica rice grains via transcriptional profiling and enzymatic activity analysis**

Wichian Sangwongchai^a,#^, Kwanjeera Wanichthanarak^b,c,#^, Ammarin In-on^b^, Supidcha Natee^a^, Chamaiporn Champasri^a^, Nutcha Sa-ingthong^a^, Diane M. Beckles^d^, Sakda Khoomrung^b,c,e^, and Maysaya Thitisaksakul^a,*^

**Supplementary Materials**

**Table S1. The number of reads before and after the filtering steps.**

|  | **Before QC** | | | **After QC** | | | |
| --- | --- | --- | --- | --- | --- | --- | --- |
| **Sample Name** | **Number of sequence (Million)** | **Unique Reads** | **Duplicate Reads** | **Unique Reads** | **Duplicate Reads** | **Number of sequence (Million)** | **%reads after QC** |
| RD57_28_1.1 | 13.5 | 5083569 | 8377661 | 4681742 | 7778981 | 12.5 | 92.57 |
| RD57_28_1.2 | 13.5 | 5213125 | 8248105 | 4788877 | 7671846 | 12.5 | 92.57 |
| RD57_28_2.1 | 11.2 | 4451934 | 6776292 | 4094276 | 6120189 | 10.2 | 90.97 |
| RD57_28_2.2 | 11.2 | 4597079 | 6631147 | 4264963 | 5949502 | 10.2 | 90.97 |
| RD57_28_3.1 | 12.3 | 4904794 | 7350217 | 4527241 | 6793460 | 11.3 | 92.38 |
| RD57_28_3.2 | 12.3 | 5239339 | 7015672 | 4900116 | 6420585 | 11.3 | 92.38 |
| RD6_28_1.1 | 12.9 | 4950384 | 7948065 | 4556123 | 7243353 | 11.8 | 91.48 |
| RD6_28_1.2 | 12.9 | 5109958 | 7788491 | 4760860 | 7038616 | 11.8 | 91.48 |
| RD6_28_2.1 | 16 | 5789499 | 10182111 | 5345832 | 9255590 | 14.6 | 91.42 |
| RD6_28_2.2 | 16 | 6016008 | 9955602 | 5598189 | 9003233 | 14.6 | 91.42 |
| RD6_28_3.1 | 12.9 | 5278076 | 7641219 | 4867286 | 7007406 | 11.9 | 91.91 |
| RD6_28_3.2 | 12.9 | 5610133 | 7309162 | 5226376 | 6648316 | 11.9 | 91.91 |
| RD57_21_1.1 | 11.4 | 3916359 | 7485584 | 3566315 | 6703541 | 10.3 | 90.07 |
| RD57_21_1.2 | 11.4 | 4046512 | 7355431 | 3714668 | 6555188 | 10.3 | 90.07 |
| RD57_21_2.1 | 15.8 | 4613834 | 11231864 | 4204485 | 10059163 | 14.3 | 90.02 |
| RD57_21_2.2 | 15.8 | 4988840 | 10856858 | 4537688 | 9725960 | 14.3 | 90.02 |
| RD57_21_3.1 | 10.9 | 4074428 | 6845806 | 3750882 | 6026465 | 9.8 | 89.53 |
| RD57_21_3.2 | 10.9 | 4353359 | 6566875 | 4021610 | 5755737 | 9.8 | 89.53 |
| RD6_21_1.1 | 13.8 | 6146643 | 7665923 | 5510365 | 6987404 | 12.5 | 90.48 |
| RD6_21_1.2 | 13.8 | 5909010 | 7903556 | 5219084 | 7278685 | 12.5 | 90.48 |
| RD6_21_2.1 | 14.7 | 5952889 | 8719121 | 5410625 | 8092969 | 13.5 | 92.04 |
| RD6_21_2.2 | 14.7 | 5590485 | 9081525 | 5032387 | 8471207 | 13.5 | 92.04 |
| RD6_21_3.1 | 11.9 | 5143597 | 6714362 | 4615380 | 6209440 | 10.8 | 91.29 |
| RD6_21_3.2 | 11.9 | 4891059 | 6966900 | 4358927 | 6465893 | 10.8 | 91.29 |
| RD57_14_1.1 | 16.7 | 4335207 | 12338865 | 4008842 | 11424418 | 15.4 | 92.56 |
| RD57_14_1.2 | 16.7 | 4441266 | 12232806 | 3988422 | 11444838 | 15.4 | 92.56 |
| RD57_14_2.1 | 15.6 | 3620802 | 11934164 | 3302428 | 11329743 | 14.6 | 94.07 |
| RD57_14_2.2 | 15.6 | 3805541 | 11749425 | 3470148 | 11162023 | 14.6 | 94.07 |
| RD57_14_3.1 | 12.9 | 3143615 | 9774602 | 2854662 | 9087536 | 11.9 | 92.44 |
| RD57_14_3.2 | 12.9 | 3280763 | 9637454 | 2981014 | 8961184 | 11.9 | 92.44 |
| RD6_14_1.1 | 13.3 | 3485182 | 9833055 | 3180044 | 9103803 | 12.3 | 92.23 |
| RD6_14_1.2 | 13.3 | 3568919 | 9749318 | 3242262 | 9041585 | 12.3 | 92.23 |
| RD6_14_2.1 | 11.9 | 3343228 | 8582578 | 2997842 | 6852040 | 9.8 | 82.59 |
| RD6_14_2.2 | 11.9 | 3417792 | 8508014 | 3072759 | 6777123 | 9.8 | 82.59 |
| RD6_14_3.1 | 13.7 | 3795455 | 9863238 | 3510762 | 9100692 | 12.6 | 92.33 |
| RD6_14_3.2 | 13.7 | 4087645 | 9571048 | 3781615 | 8829839 | 12.6 | 92.33 |
| RD57_7_1.1 | 13.8 | 3597137 | 10192843 | 3285101 | 9372512 | 12.7 | 91.79 |
| RD57_7_1.2 | 13.8 | 3820425 | 9969555 | 3482118 | 9175495 | 12.7 | 91.79 |
| RD57_7_2.1 | 11.9 | 3259820 | 8688986 | 2953647 | 7974734 | 10.9 | 91.46 |
| RD57_7_2.2 | 11.9 | 3425534 | 8523272 | 3074319 | 7854062 | 10.9 | 91.46 |
| RD57_7_3.1 | 12.3 | 4057227 | 8224341 | 3704280 | 7577789 | 11.3 | 91.86 |
| RD57_7_4.2 | 12.3 | 4258448 | 8023120 | 3907045 | 7375024 | 11.3 | 91.86 |
| RD6_7_1.1 | 13.2 | 4165458 | 9016900 | 3809511 | 8371613 | 12.2 | 92.40 |
| RD6_7_1.2 | 13.2 | 4446083 | 8736275 | 4078653 | 8102471 | 12.2 | 92.40 |
| RD6_7_2.1 | 11.7 | 3358170 | 8312081 | 3002928 | 4877160 | 7.9 | 67.52 |
| RD6_7_2.2 | 11.7 | 3438142 | 8232109 | 3117282 | 4762806 | 7.9 | 67.52 |
| RD6_7_3.1 | 13.1 | 4725206 | 8398906 | 4385394 | 7888214 | 12.3 | 93.52 |
| RD6_7_32 | 13.1 | 4879790 | 8244322 | 4512890 | 7760718 | 12.3 | 93.52 |

**Table S2. Summary of read alignment to the *Oryza sativa* indica reference genome.**

| **Sample** | **%Aligned** | **PE mapped uniquely (reads)** | **PE multimapped (reads)** | **PE neither mate aligned (reads)** |
| --- | --- | --- | --- | --- |
| RD57_28_1 | 93.9 | 11,148,729 (89.5%) | 546,757 (4.4%) | 765,237 (6.1%) |
| RD57_28_2 | 92.5 | 9,029,299 (88.4%) | 421,666 (4.1%) | 763,500 (7.5%) |
| RD57_28_3 | 93.7 | 10,108,914 (89.3%) | 503,547 (4.4%) | 708,240 (6.3%) |
| RD6_28_1 | 93.6 | 10,549,754 (89.4%) | 494,980 (4.2%) | 754,742 (6.4%) |
| RD6_28_2 | 92.8 | 12,775,149 (87.5%) | 770,307 (5.3%) | 1,055,966 (7.2%) |
| RD6_28_3 | 93.6 | 10,604,100 (89.3%) | 513,136 (4.3%) | 757,456 (6.4%) |
| RD57_21_1 | 93.3 | 9,082,159 (88.4%) | 503,396 (4.9%) | 684,301 (6.7%) |
| RD57_21_2 | 92.7 | 12,488,106 (87.6%) | 740,232 (5.2%) | 1,035,310 (7.3%) |
| RD57_21_3 | 93.1 | 8,630,714 (88.3%) | 468,867 (4.8%) | 677,766 (6.9%) |
| RD6_21_1 | 89.3 | 10,551,296 (84.4%) | 611,273 (4.9%) | 1,335,200 (10.7%) |
| RD6_21_2 | 90.5 | 11,411,304 (84.5%) | 811,051 (6.0%) | 1,281,239 (9.5%) |
| RD6_21_3 | 89.3 | 9,171,226 (84.7%) | 493,328 (4.6%) | 1,160,266 (10.7) |
| RD57_14_1 | 91.7 | 13,304,481 (86.2%) | 847,912 (5.5%) | 1,280,867 (8.3%) |
| RD57_14_2 | 92 | 12,693,081 (86.7%) | 762,548 (5.2%) | 1,176,542 (8.0%) |
| RD57_14_3 | 91.8 | 10,271,059 (86.0%) | 693,476 (5.8%) | 977,663 (8.2%) |
| RD6_14_1 | 90.1 | 10,482,628 (85.3%) | 584,692 (4.8%) | 1,216,527 (9.9%) |
| RD6_14_2 | 90.7 | 8,481,658 (86.1%) | 455,777 (4.6%) | 912,447 (9.3%) |
| RD6_14_3 | 90.4 | 10,819,792 (85.8%) | 579,679 (4.6%) | 1,211,983 (9.6%) |
| RD57_7_1 | 92.8 | 10,970,803 (86.7%) | 772,491 (6.1%) | 914,319 (7.2%) |
| RD57_7_2 | 92.4 | 9,495,267 (86.9%) | 598,141 (5.5%) | 834,973 (7.6%) |
| RD57_7_3 | 93 | 9,779,875 (86.7%) | 711,202 (6.3%) | 790,992 (7.0%) |
| RD6_7_1 | 91.9 | 10,519,422 (86.4%) | 678,554 (5.6%) | 983,148 (8.1%) |
| RD6_7_2 | 92.5 | 6,822,184 (86.6%) | 470,665 (6.0%) | 587,239 (7.5%) |
| RD6_7_3 | 92.6 | 10722951 (87.4%) | 646025 (5.3%) | 904632 (7.4%) |

**Table S3. Summary of the completeness of the transcript structures.**

| **Feature** | **Number of BUSCO group** |
| --- | --- |
| Complete and single-copy BUSCOs (S) | 319 (6.5%) |
| Complete and duplicated BUSCOs (D) | 4,465 (91.2) |
| Fragmented BUSCOs (F) | 54 (1.1%) |
| Missing BUSCOs (M) | 58 (1.2%) |
| Total | 4896 |

**Table S4. Primer sequences used in the study.**

| **Genes**  **(accession ID)** | **Protein names** |  | **Primer sequences (5’-3’)** |
| --- | --- | --- | --- |
|  |  |  |  |
| *OsAGPSI*  (AY028315) | ADP-glucose pyrophosphorylase small subunit 1 | F | TATGACCGTTCTGCCGCAAT |
|  |  | R | GAGTCCTCTATCACTGCGCC |
| *OsBEI*  (AF136268) | Starch branching enzyme 1 | F | ATGCATTGCCTATGCCGAGA |
|  |  | R | ATGCCACGGTTGATGGTAGG |
| *OsGluD1* (NM_001409963) | Glutelin D1 | F | AAGACAGAGCGACCAAGCTC |
|  |  | R | ATGTGCAACACTAGCCGGAA |
| *OsAct1* (KX302608) | Actin 1 | F | TGTATGCCAGTGGTCGTACCA |
|  |  | R | CCAGCAAGGTCGAGACGAA |
| *OsEF-1α*  (XM_015774317) | Eukaryotic elongation factor 1-alpha | F | GGAGAAGACGCACATCAACA |
|  |  | R | GGCTTCCTTCTCGAACCTCT |

**Table S5. Summary of significant correlation pairs in co-expression analysis**

|  | **Pattern 40**  Total correlations (positive/negative;  co-expression network) | **Pattern 45**  Total correlations (positive/negative;  co-expression network) |
| --- | --- | --- |
| 7 DPA | 31,959 (26,715/5,244; 97) | 58,815 (47,442/11,373; 58) |
| 14 DPA | 39,686 (32,040/7,646; 53) | 44,194 (36,520/7,674; 5) |
| 21 DPA | 46,099 (43,564/2,535; 204) | 34,725 (32,431/2,294; 18) |
| 28 DPA | 1,880 (1,364/516; 87) | 2,622 (2,042/580; 10) |

**Table S6. Summary of correlation values of selected co-expressed genes in Fig. 7**

| **Correlation pairs** | **Correlation coefficient** | **p-value** |
| --- | --- | --- |
| DAPB3-like x GluA-1.2 | 1.000 | < 0.001 |
| DAPB3-like x GluA-1.3 | 0.943 | 0.005 |
| GBSSI x PPA1-like | 0.943 | 0.005 |
| ASK4-like x GluA-1.3 | 0.941 | 0.005 |
| ASK4-like x GluD-1 | 0.941 | 0.005 |
| HBT9-like x GluA-1.3 | 0.943 | 0.005 |
| HBT9-like x GluD-1 | 0.943 | 0.005 |
| RCAR3-like x AgpS2b | 0.943 | 0.005 |
| RCAR3-like x AgpL2 | 0.943 | 0.005 |
| RCAR3-like x BEIIb | 0.943 | 0.005 |
| RCAR3-like x PUL | 1.000 | < 0.001 |
| CRA1-like x GluA-1.2 | 1.000 | < 0.001 |
| CRA1-like x GluA-1.3 | 1.000 | < 0.001 |
| CRA1-like x GluD-1 | 0.943 | 0.005 |
| GBSSI x DUF630\|DUF632 | 0.941 | 0.005 |

**Table S7. Summary of docking models between each TF and its corresponding genes**

| **TF** | **Gene** | **Osl_id** | **Gene_id** | **Motif_id** | **score** | **irmsd** | **lrmsd** | **fnat** |
| --- | --- | --- | --- | --- | --- | --- | --- | --- |
| bZIP10 | AgpL2 | OsI_02968 | BGIOSGA004052 | M1 | -603.476 | 0 | 0 | 1 |
| bZIP10 | AgpL2 | OsI_02968 | BGIOSGA004052 | M45 | -592.847 | 0 | 0 | 1 |
| bZIP10 | AgpL3 | OsI_13419 | BGIOSGA009855 | M55 | -574.623 | 0 | 0 | 1 |
| bZIP10 | BEI | OsI_24520 | BGIOSGA020506 | M85 | -588.807 | 0 | 0 | 1 |
| bZIP10 | DPEII | OsI_27219 | BGIOSGA026328 | M93 | -493.435 | 0 | 0 | 1 |
| bZIP10 | GluA-1.1 | OsI_03830 | BGIOSGA004498 | M51 | -528.182 | 0 | 0 | 1 |
| bZIP10 | GluA-1.2 | OsI_03835 | BGIOSGA004500 | M1 | -603.476 | 0 | 0 | 1 |
| bZIP10 | GluA-1.3 | OsI_33479 | BGIOSGA031970 | M1 | -603.476 | 0 | 0 | 1 |
| bZIP10 | GluA-1.3 | OsI_33479 | BGIOSGA031970 | M11 | -576.462 | 0 | 0 | 1 |
| bZIP10 | GluA-1.3 | OsI_33479 | BGIOSGA031970 | M13 | -567.33 | 0 | 0 | 1 |
| bZIP10 | GluA-1.3 | OsI_33479 | BGIOSGA031970 | M9 | -577.309 | 0 | 0 | 1 |
| bZIP10 | GluD-1 | OsI_06564 | BGIOSGA006784 | M16 | -586.369 | 0 | 0 | 1 |
| bZIP10 | GluD-1 | OsI_06564 | BGIOSGA006784 | M19 | -508.346 | 0 | 0 | 1 |
| bZIP10 | ISA1 | OsI_29923 | BGIOSGA026650 | M105 | -621.515 | 0 | 0 | 1 |
| bZIP10 | PHOL | OsI_13605 | BGIOSGA009780 | M60 | -561.866 | 0 | 0 | 1 |
| bZIP10 | PUL | OsI_14800 | BGIOSGA015875 | M69 | -584.447 | 0 | 0 | 1 |
| bZIP10 | SSI | OsI_21770 | BGIOSGA021861 | M1 | -603.476 | 0 | 0 | 1 |
| bZIP10 | SSI | OsI_21770 | BGIOSGA021861 | M29 | -555.288 | 0 | 0 | 1 |
| bZIP10 | SSI | OsI_21771 | BGIOSGA021860 | M32 | -499.509 | 0 | 0 | 1 |
| bZIP10-bZIP44 | AgpL2 | OsI_02968 | BGIOSGA004052 | M45 | -647.791 | 0 | 0 | 1 |
| bZIP10-bZIP44 | AgpL2 | OsI_02969 | BGIOSGA004053 | M47 | -609.095 | 0 | 0 | 1 |
| bZIP10-bZIP44 | BEI | OsI_24520 | BGIOSGA020506 | M80 | -576.78 | 0 | 0 | 1 |
| bZIP10-bZIP44 | BEIIa | OsI_15790 | BGIOSGA015140 | M72 | -581.811 | 0 | 0 | 1 |
| bZIP10-bZIP44 | GluA-1.1 | OsI_03830 | BGIOSGA004498 | M49 | -614.93 | 0 | 0 | 1 |
| bZIP10-bZIP44 | GluA-1.1 | OsI_03830 | BGIOSGA004498 | M51 | -574.231 | 0 | 0 | 1 |
| bZIP10-bZIP44 | GluA-1.2 | OsI_03835 | BGIOSGA004500 | M1 | -665.137 | 0 | 0 | 1 |
| bZIP10-bZIP44 | GluA-1.2 | OsI_03835 | BGIOSGA004500 | M7 | -575.063 | 0 | 0 | 1 |
| bZIP10-bZIP44 | GluA-1.3 | OsI_33479 | BGIOSGA031970 | M1 | -665.137 | 0 | 0 | 1 |
| bZIP10-bZIP44 | GluD-1 | OsI_06564 | BGIOSGA006784 | M16 | -587.304 | 0 | 0 | 1 |
| bZIP10-bZIP44 | GluD-1 | OsI_06564 | BGIOSGA006784 | M17 | -661.836 | 0 | 0 | 1 |
| bZIP10-bZIP44 | GluD-1 | OsI_06564 | BGIOSGA006784 | M19 | -533.965 | 0 | 0 | 1 |
| bZIP10-bZIP44 | PHOL | OsI_13605 | BGIOSGA009780 | M60 | -593.52 | 0 | 0 | 1 |
| bZIP10-bZIP44 | SSI | OsI_21770 | BGIOSGA021861 | M1 | -665.137 | 0 | 0 | 1 |
| bZIP10-bZIP44 | SSII-3(SSIIa) | OsI_22256 | BGIOSGA022586 | M39 | -601.867 | 0 | 0 | 1 |
| bZIP10-bZIP44 | SSII-3(SSIIa) | OsI_22256 | BGIOSGA022586 | M41 | -551.257 | 0 | 0 | 1 |

**
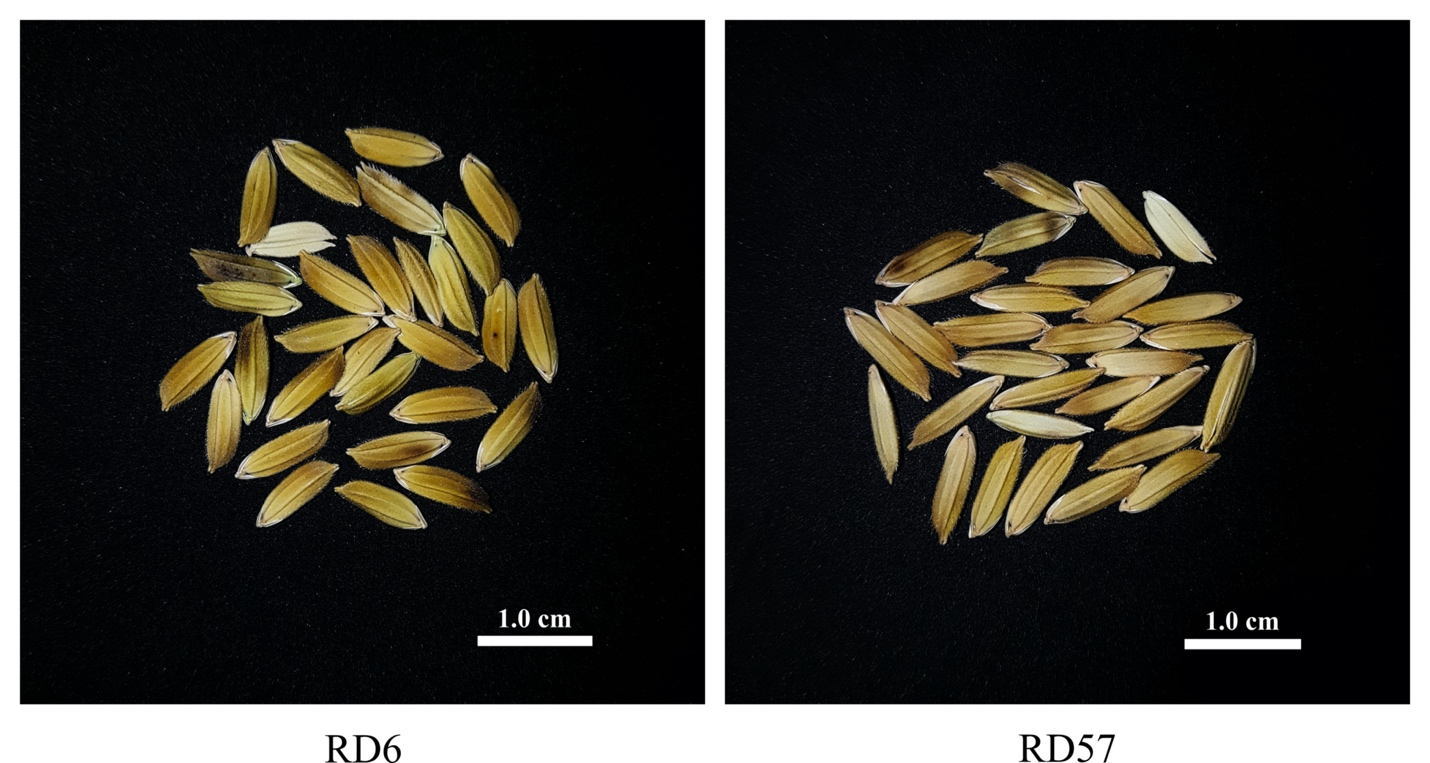
**

**Fig. S1.** Representation of the grain appearances of two rice cultivars at the maturity stage


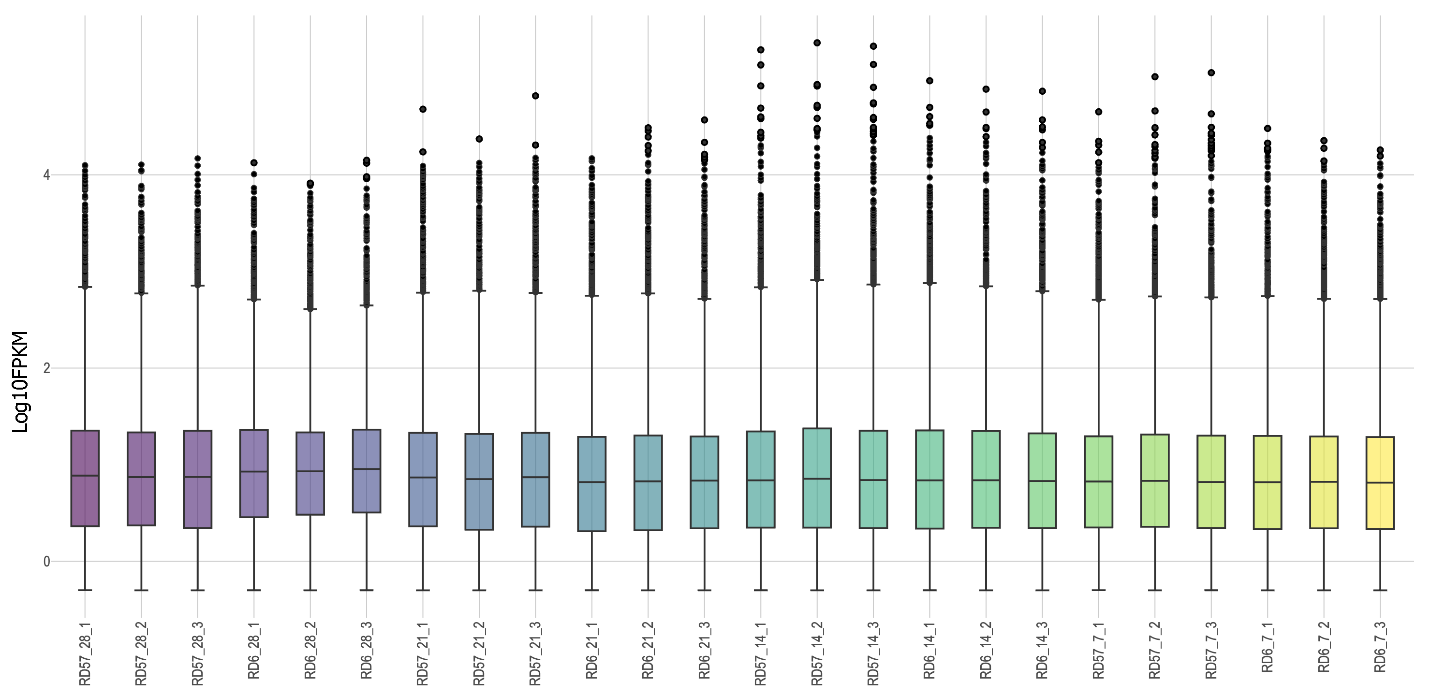


**Fig. S2.** Boxplot of FPKM values for each sample. In each boxplot, the upper and lower bounds represent the first and fourth quartiles, with the middle line represents the median. The x-axis displays rice genotypes at each DPA and their three replicates, while the y-axis shows the log10(FPKM). This plot demonstrates that the FPKM values are comparable across the samples.

**
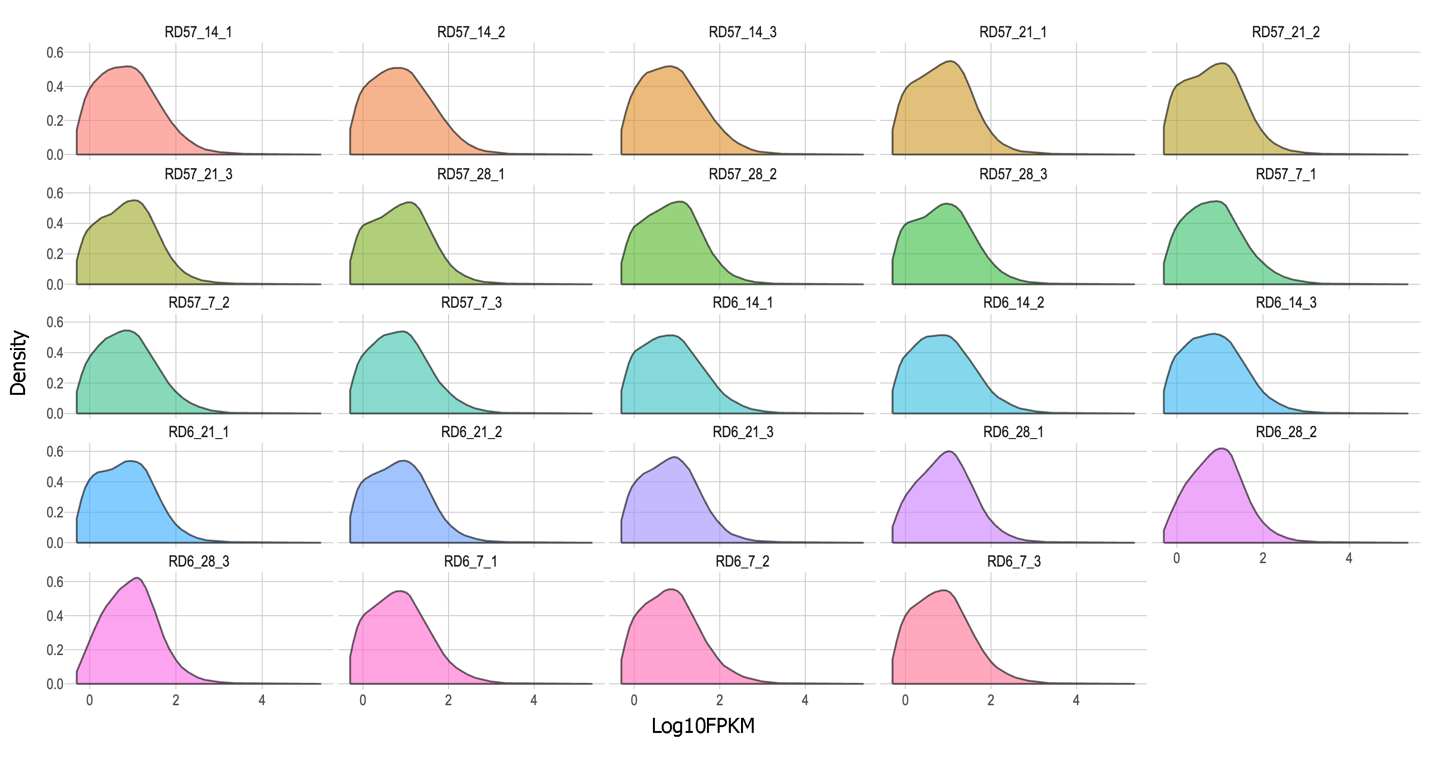
**

**Fig. S3.** Gene expression distribution for each sample. In this study, the distribution of gene expression follows a negative binomial distribution.

**
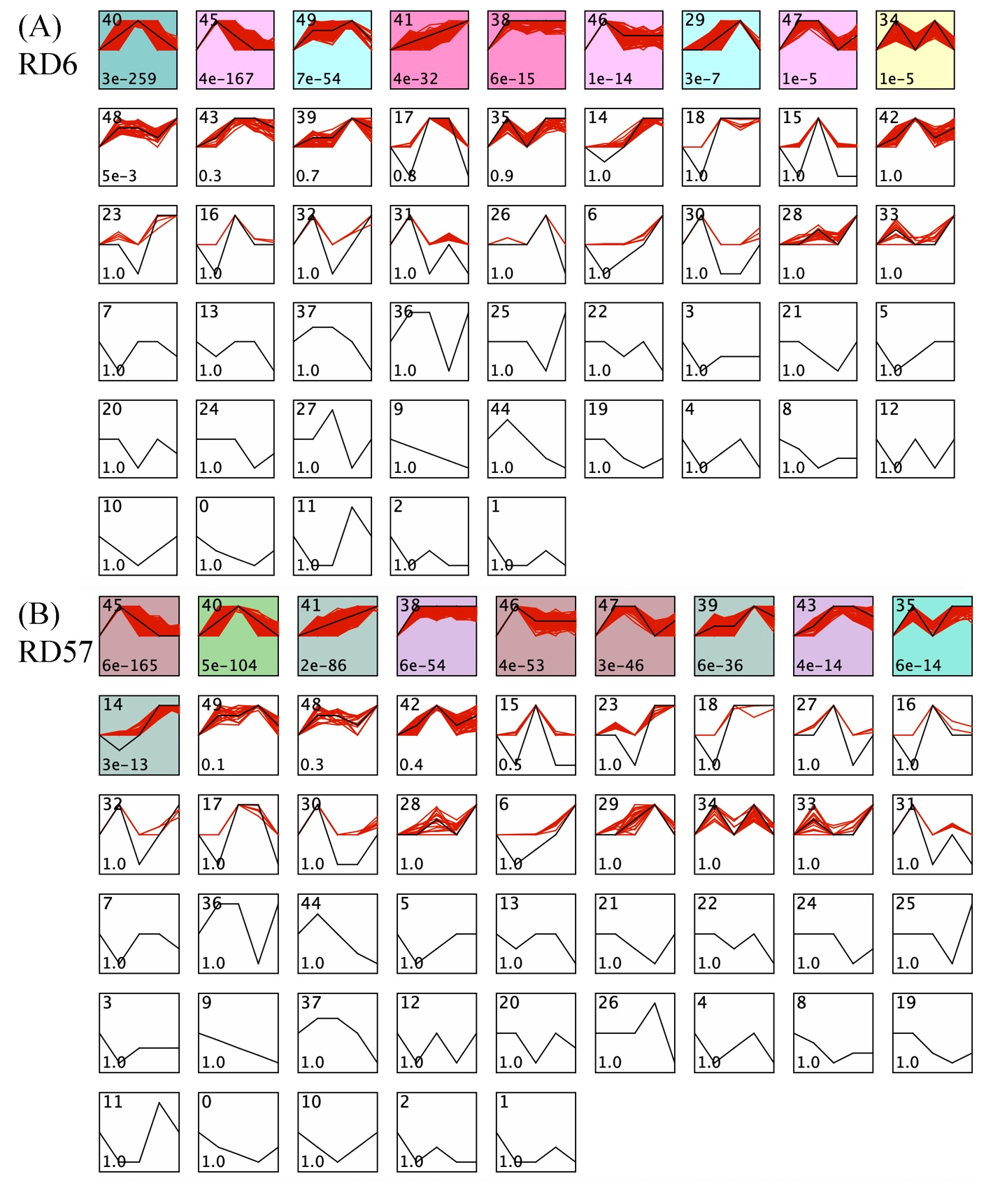
**

**Fig. S4.** Temporal gene expression patterns of DEGs. (A) Identified patterns for RD6 and (B) identified patterns for RD57. Each box illustrates the temporal expression profiles of RD6 and RD57, where the pattern names is in the top-left and the corrected p-value in the bottom-left. Profile patterns are ordered based on their significance. A box with colored background indicates a significant temporal expression profile pattern. Boxes with the same color belong to the same cluster, as identified by K-means clustering.

**
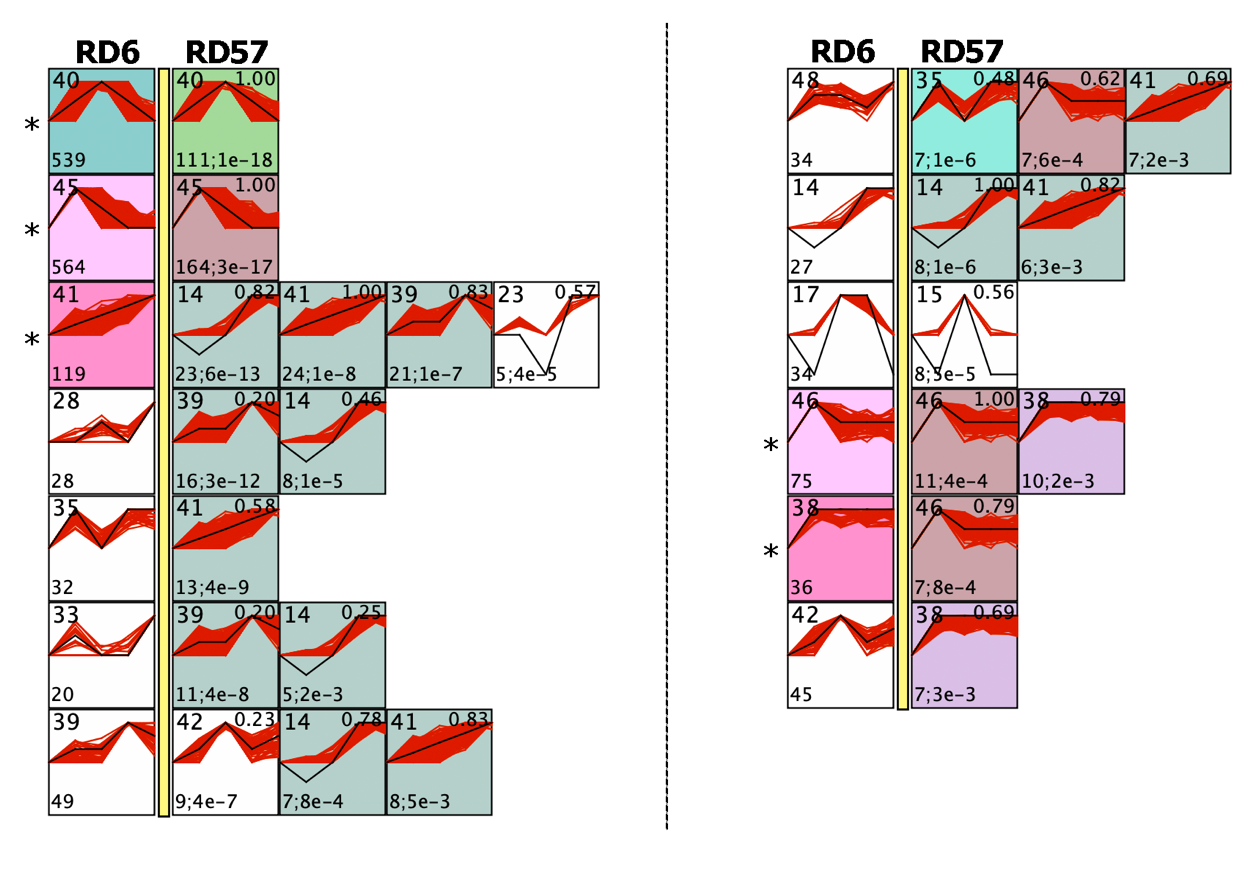
**

**Fig. S5.** Comparisons of temporal expression profiles between RD57 and RD6. Profile patterns showing significantly correlated temporal profiles between RD57 and RD6 are marked with an asterisk (*). Each box represents the temporal expression profiles of RD57 and RD6, with the pattern names in the top-left corner. A box with colored background indicates a significant temporal expression pattern. The correlation coefficients, p-values, and the number of overlapping genes are displayed in the top-right and bottom-left corners of the RD57 box, respectively.


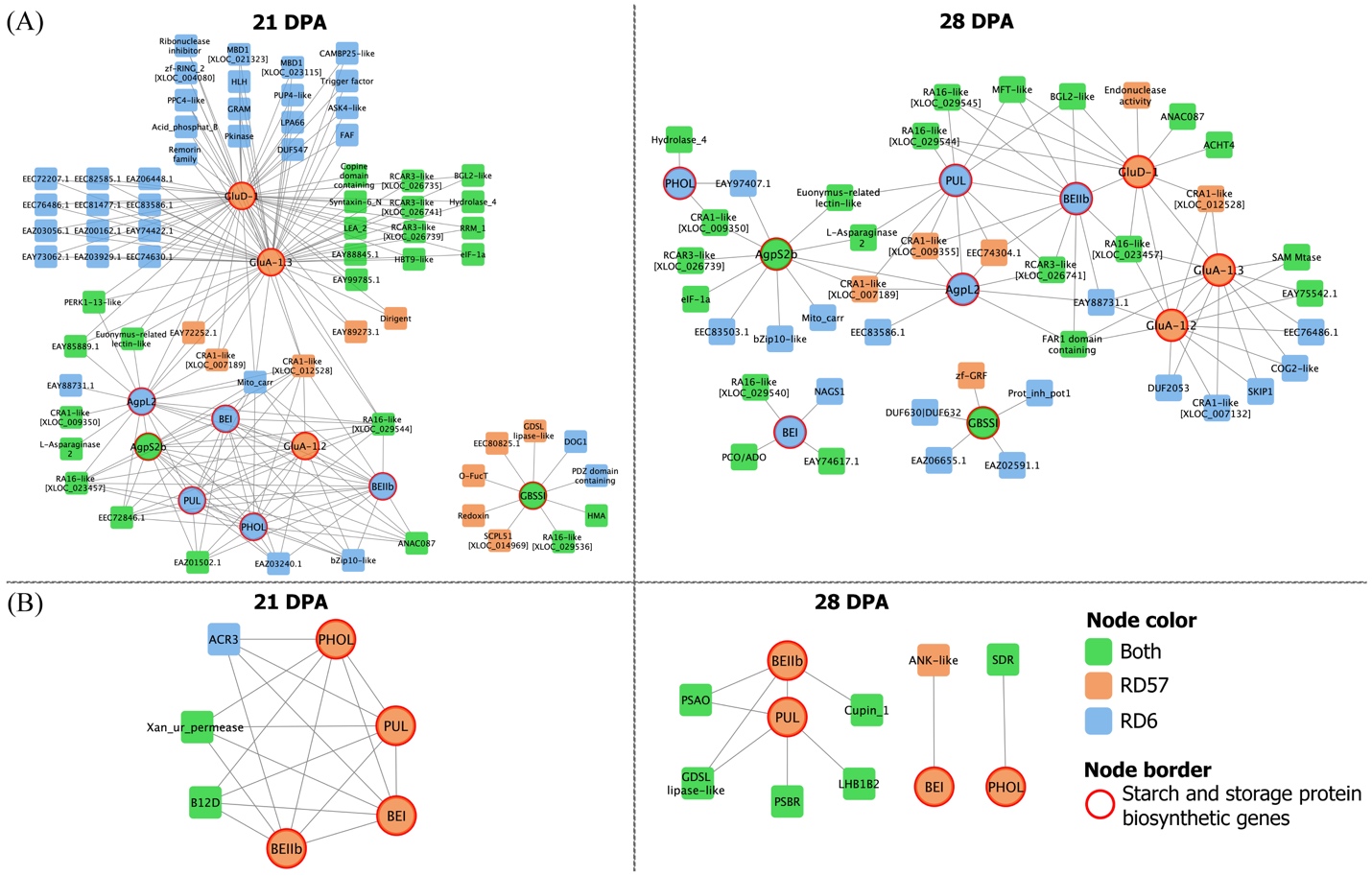


**Fig. S6.** Co-expression networks of genes involved in starch biosynthesis. (A) Selected genes from Pattern 40 at 21 and 28 DPA. (B) Selected genes from Pattern 45 at 21 and 28 DPA. The color of the nodes corresponds to the rice genotypes, with green representing genes common to both RD6 and RD57, blue for RD6-specific genes, and orange for RD57-specific genes. Starch markers are represented as nodes with a red border.
